# Study on Chemical Modifications of Glutathione by Cold Atmospheric Pressure Plasma (Cap) Operated in Air in the Presence of Fe(II) and Fe(III) Complexes

**DOI:** 10.1038/s41598-019-53538-y

**Published:** 2019-12-02

**Authors:** Dariusz Śmiłowicz, Friederike Kogelheide, Katharina Stapelmann, Peter Awakowicz, Nils Metzler-Nolte

**Affiliations:** 1 Inorganic Chemistry I - Bioinorganic Chemistry, Ruhr University Bochum, 44780 Bochum, Germany; 2 Institute for Electrical Engineering and Plasma Technology, Ruhr University Bochum, 44780 Bochum, Germany; 3 Department of Nuclear Engineering, North Carolina State University, Raleigh, North Carolina 27695 USA

**Keywords:** Chemical modification, Iron, Biomedical engineering

## Abstract

Cold atmospheric pressure plasma is an attractive new research area in clinical trials to treat skin diseases. However, the principles of plasma modification of biomolecules in aqueous solutions remain elusive. It is intriguing how reactive oxygen and nitrogen species (RONS) produced by plasma interact on a molecular level in a biological environment. Previously, we identified the chemical effects of dielectric barrier discharges (DBD) on the glutathione (GSH) and glutathione disulphide (GSSG) molecules as the most important redox pair in organisms responsible for detoxification of intracellular reactive species. However, in the human body there are also present redox-active metals such as iron, which is the most abundant transition metal in healthy humans. In the present study, the time-dependent chemical modifications on GSH and GSSG in the presence of iron(II) and iron(III) complexes caused by a dielectric barrier discharge (DBD) under ambient conditions were investigated by IR spectroscopy, mass spectrometry and High Performance Liquid Chromatography (HPLC). HPLC chromatograms revealed one clean peak after treatment of both GSH and GSSH with the dielectric barrier discharge (DBD) plasma, which corresponded to glutathione sulfonic acid GSO_3_H. The ESI-MS measurements confirmed the presence of glutathione sulfonic acid. In our experiments, involving either iron(II) or iron(III) complexes, glutathione sulfonic acid GSO_3_H appeared as the main oxidation product. This is in sharp contrast to GSH/GSSG treatment with DBD plasma in the absence of metal ions, which gave a wild mixture of products. Also interesting, no nitrosylation of GSH/GSSG was oberved in the presence of iron complexes, which seems to indicate a preferential oxygen activation chemistry by this transition metal ion.

## Introduction

Cold atmospheric plasma (CAP) is used in clinical trials for skin disinfection^1^, wound healing^2^ and the treatment of other skin disorders^3^. Among various plasma techniques, dielectric barrier discharge (DBD) is in the spotlight due to its safe treatment mode, its possibility of direct plasma ignition in ambient air and its ability to treat large surfaces uniformly^4,5^. Nowadays, its application is extended into several diverse fields, such as medicine^6,7^, environmental science^8,9^ or agriculture^10,11^. The effects of DBD treatments on promotion of cancer cells apoptosis^12,13^, proliferation of osteoblasts^14,15^, inactivation of enzymes^16,17^ and oxidative conversion of organic molecules^18,19^ are deeply investigated. CAP is being consider as a promising alternative approach compared to conventional anticancer therapy, due to the cytotoxic activity towards cancer cells, especially skin cancer^20,21^. DBD plasma is owing such a broad range of application to the ample amount of reactive oxygen species (ROS) and reactive nitrogen species (RNS) generated during the treatment^22,23^. Simultaneously, the impact on normal cells and treatment parameters are being established to provide the safety of plasma for biological applications^24,25^. However, it is most challenging to understand and control the interactions of plasma with cellular components of living cells and biomolecules, such as sugars, lipids, vitamins and amino acids, which are the major chemical species in cell culture medium. Understanding of the reaction intermediates and products of these chemicals under non-thermal plasma is required to promote and further optimize the development of clinical applications of plasma treatment. Takai *et al*. studied in detail the chemical modification of 20 naturally occurring amino acids caused by plasma treatment in aqueous solution^26^. The study revealed that sulfur-containing and aromatic amino acids were preferentially degraded by plasma treatment through the hydroxylation and nitration of aromatic rings in tyrosine, phenylalanine and tryptophan; sulfonation and disulfide linkage formation of thiol groups in cysteine; sulfoxidation of methionine and amidation and ring-opening of five-membered rings in histidine and proline. Li *et al*. investigated the influence of non-thermal plasma generated by using a DBD on solutions of ribose, glucose, and sucrose in water and phosphate buffer^27^. The results showed the time-dependent decomposition of these sugars to formic acid, glycolic acid, glyceric acid, tartronic acid, tartaric acid, acetic acid, and oxalic acid after direct exposure to plasma. Smith *et al*. determined also the potency of non-thermal atmospheric pressure plasma as an alternative to advanced oxidation processes for antibiotic removal from water^28^. The antibiotic, ampicillin, was completely eliminated during five minutes after air-plasma treatment giving ampicillin sulfoxide as the main product. Zhang *et al*. have investigated the effects of a DBD on lactate dehydrogenase (LDH), an important sugar metabolic enzyme^29^. Their study showed the inactivation of LDH enzyme from plasma-induced modification of the secondary molecular structure as well as polymerization of the peptide chains after treatment for 300 s. Choi *et al*. have treated lysozyme enzyme with two types of plasma sources, a DBD and an atmospheric pressure plasma jet (APPJ) using N_2_ and air as the feeding gases for 8 min and 12 min^30^. The results revealed the structural changes in loop 3, loop 6 and in the substrate binding site of lysozyme. Park *et al*. have investigated the influence of ROS and RNS generated from dielectric barrier discharge plasma in aqueous solution on the conformations of two model proteins, hemoglobin (Hb) and myoglobin (Mb)^31^. The CD data revealed that the percentage of *α*-helices decreases and the percentage of *β*-sheet increases as the treatment continues from 1 min to 2 and 4 min. Yong *et al*. confirmed the mechanism of green discolouration of myoglobin induced by atmospheric pressure plasma (APP)^32^. The treatment of myoglobin in phosphate buffer by APP for 20 min produced nitrite ions along with the formation of nitrimyoglobin. Finally, Kim *et al*. investigated the effects of non-thermal plasma on rheological characteristics of red blood cells (RBC)^33^. This study showed that plasma exposure times longer than 2 min resulted in hemorheological alterations such as hemolytic activity, elongation index and higher aggregation index than in the untreated RBC samples. All these studies of plasma treatments were performed for samples in aqueous solutions and all organic molecues decomposed to *smaller* chemical species.

**Figure 1:**
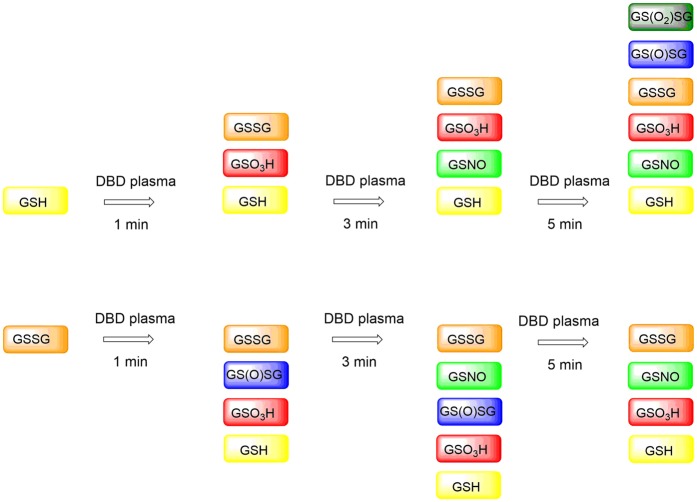
Chemical modifications of GSH and GSSG after different times of plasma treatment^38^.

In our research, we focus on the impact of a DBD on glutathione (GSH) and glutathione disulphide (GSSG). GSH plays a key role in cell resistance to oxidative damage to tissues and cells by minimizing the ROS concentration^34^. Upon the onset of oxidative stress, two GSH molecules are oxidized to glutathione disulphide (GSSG), which can be reversibly reduced to GSH by GSSG reductase^35^. GSH occurs at ~1–2 mM concentrations in most human cells, which together with its oxidized counterpart, glutathione disulphide (GSSG) makes it the most abundant intracellular redox couple^36,37^. Previously^38^, we identified the time-dependent chemical modifications on GSH and GSSG caused by DBD treatment under ambient conditions. We found that besides glutathione sulfonic acid (GSO_3_H) and S-nitrosoglutathione (GSNO) as the main products, a wide variety of oxidation species was formed as presented in Fig. 1. Increasing treatment time resulted in additional by-products, which could not be identified. The extensive mixture of different oxidation species made this system difficult to monitor (see Fig. 1).

**Figure 2:**
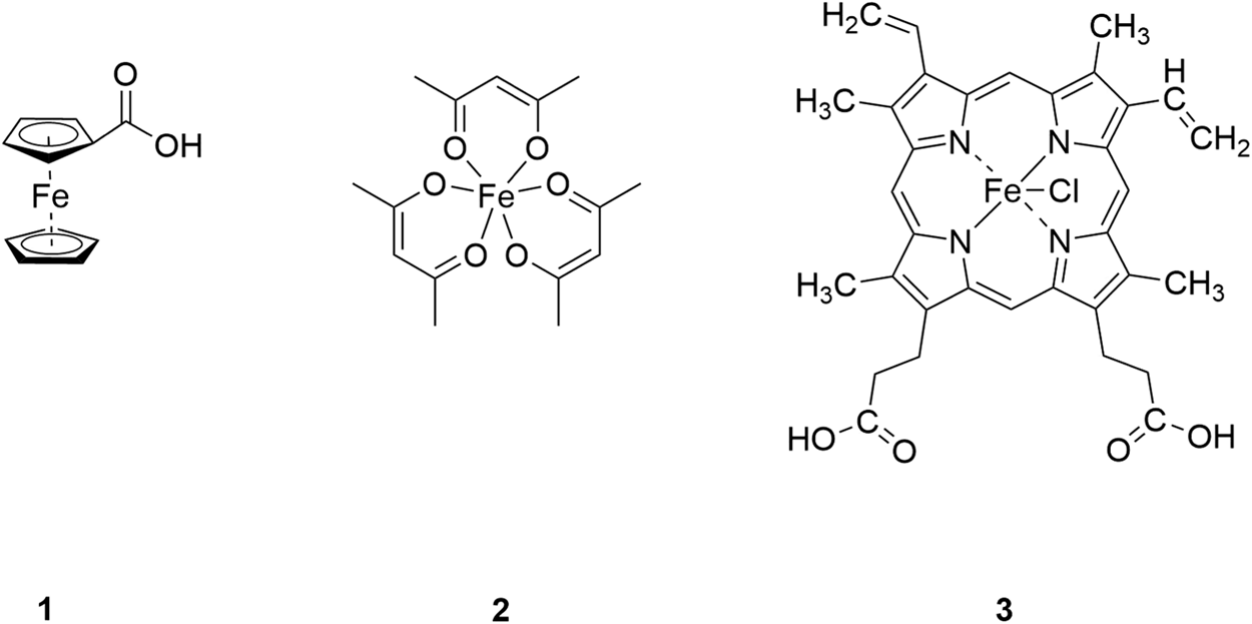
Structures of iron complexes: ferrocenecarboxylic acid (**1**), iron(III) acetylacetonate (**2**) and hemin (**3**).

It is well-known that transition metals like manganese, iron, cobalt, copper or zinc have noticeable effects on human health. Indeed, all these transition metals are essential trace elements for humans. Among them, iron is the most abundant in healthy humans and participates in numerous vital functions such as oxygen transport, DNA synthesis, and electron transfer. Also, iron is essential in maintaining the function of the central nervous system^39^. As a redox-active transition metal, iron occurs at physiological pH in reduced ferrous form (Fe^2+^), which can be rapidly oxidized to the ferric (Fe^3+^) form^40–42^. To further develop our understanding of the impact of plasma treatment on GSH and GSSG as the central cellular redox system, we utilized the iron(II) complex ferrocenecarboxylic acid (complex **1**) and the iron(III) complexes iron(III) acetylacetonate (Fe(acac)_3_) (complex **2**) and chloro(protoporphyrinato)iron(III) (hemin) (complex **3** Fig. 2) to mimic the environment in human cells, and in particular the presence and possible influence of further ubiquitous redox-active cellular components. We investigated: (i) the influence of plasma on iron(II) and iron(III) complexes and subsequently (ii) chemical modifications on GSH and GSSG caused by DBD plasma in the presence of these iron complexes.

## Results

### Stability experiments

In a first step, the stability of all iron complexes in distilled water was monitored by ESI-MS. Electrospray ionization mass spectrometry (ESI-MS) was applied to investigate the mass of the desired compounds. The complex **1**, ionized in positive as well as in negative mode, gave signals corresponding to the [M + H]^+^ at *m*/*z* = 230 and [M − H]^−^ at *m/z* = 299. The mass spectrum of complex **2** displayed one signal with moderate intensity corresponding to the sodium adduct [M + Na]^+^ at *m/z* = 376 and the another signal with high intensity corresponding to the [M − L]^+^ at *m/z* = 254, indicating the loss of one acetyloacetone ligand. The spectrum of complex **3** exhibits one signal at *m/z* = 616 corresponding to the [M-Cl]^+^ moiety. All iron complexes showed the same, stable ionization pattern after 1, 3, 5 and 20 min. The *m/z* values are consistent with the expected isotopic mass distribution pattern. The full-scan mass spectra are available in the ESI†.

### Influence of plasma on iron complexes

In a second step the time-dependent impact of DBD treatment on the three iron complexes was investigated. Treatment of complex **1** with the DBD for 1 min already caused a loss of a H_2_O molecule giving the signal at *m/z* = 213 corresponding to [M − OH_2_]^+^. The molecular ion peak [M + H]^+^ was also found at *m/z* = 231 with moderate intensity. Extending the treatment time to 3 min as well as to 5 min caused no changes in the fragmentation pathway. The mass spectrum of complex **2** treated with plasma for 1 min displayed the same ionization pattern with signals at *m/z* = 254 and at *m/z* = 376 corresponding to the [M − L]^+^ and [M + Na]^+^, as spectrum for the untreated complex. However, the fragmentation pattern changed significantly after 3 min of plasma treatment. The signal corresponding to the sodium adduct disappeared and a new signal at *m/z* = 282 with high intensity appeared, which was assigned to complex **2** with partial loss of one ligand. The second signal remained unchanged at *m/z* = 254 as for the untreated samples. The spectrum after 5 min of treatment showed the same fragmentation pattern as the spectrum after 3 min. The spectrum for complex **3** after 1 and 3 min showed the same ionization like the spectrum of the untreated samples. The only change was observed after 5 min of plasma treatment. The MS spectrum showed three signals with moderate intensity. Despite the parent signal at *m/z* = 616 corresponding to the [M − Cl]^+^ moiety, two other indicative signals at *m/z* = 557 and at *m/z* = 506 were observed. This ionization pattern unveiled the partial degradation of the organic ligand by loss of two carboxylic acid and two CH_2_=CH_2_ groups. The above results confirm that complexes **1** and **3** survived plasma treatment even after 5min of treatment, while complex **2** decomposed after 3 min losing one ligand. The *m/z* values are consistent with the expected isotopic mass distribution pattern. The full-scan mass spectra are available in the ESI†.

**Figure 3:**
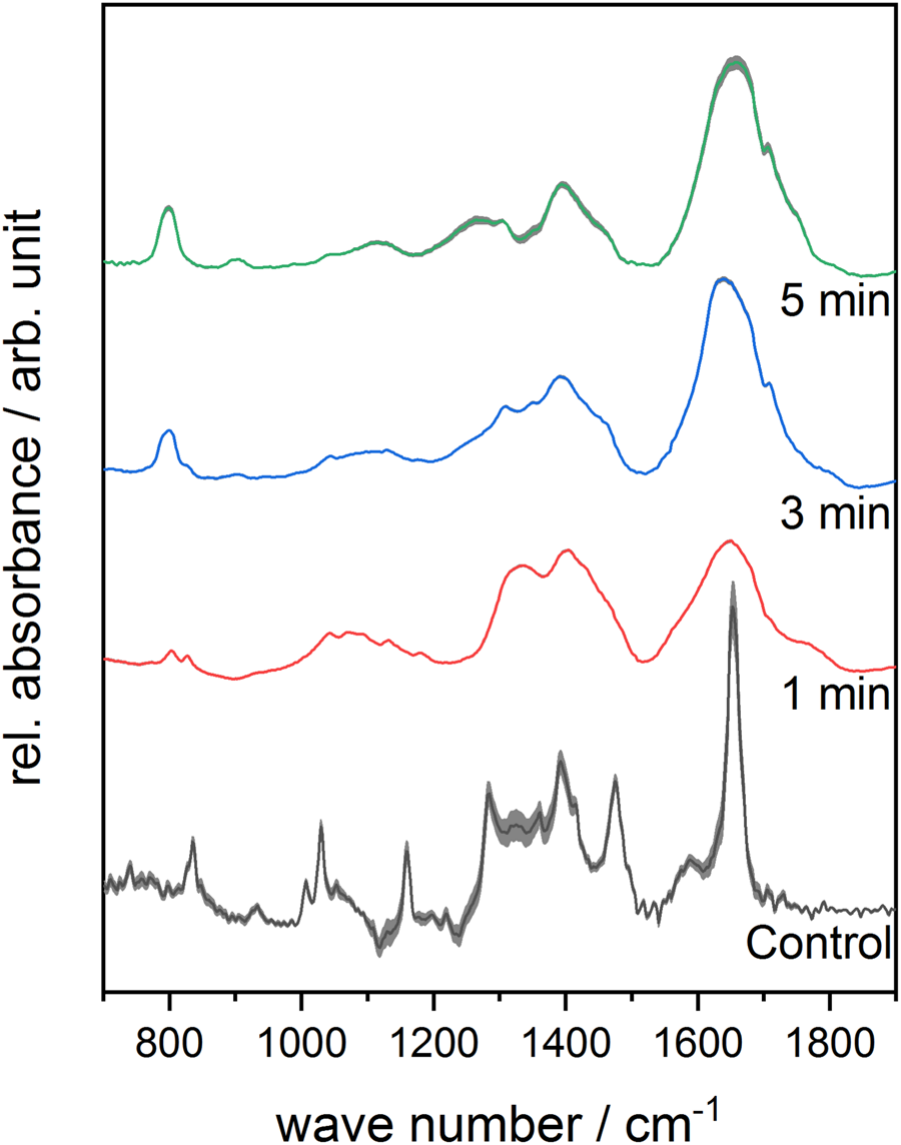
Mean FTIR-spectra of plasma-treated Complex **1** in the range of 700–1900 cm^−1^ as a function of different treatment times. Standard deviation of the mean is shown as grey area at each graph.

**Figure 4:**
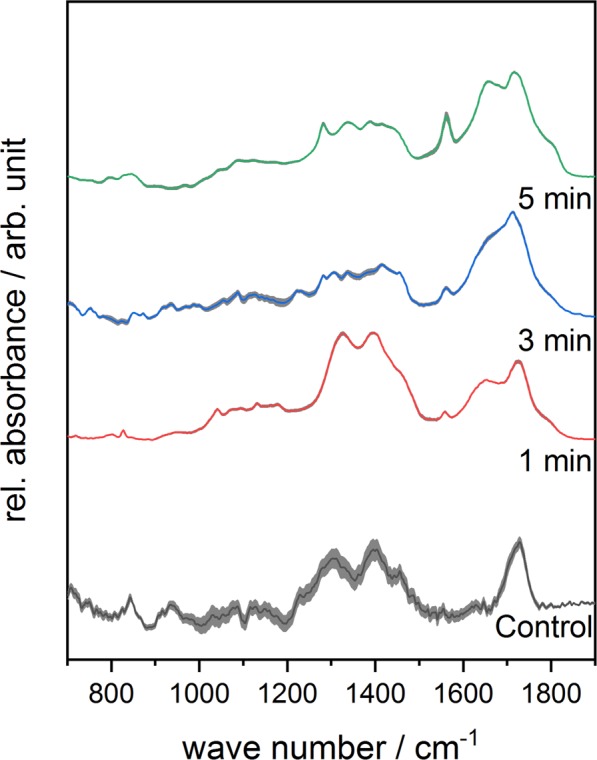
Mean FTIR-spectra of plasma-treated Complex **3** in the range of 700–1900 cm^−1^ as a function of different treatment times. Standard deviation of the mean is shown as grey area at each graph.

The IR-spectra of untreated and treated complex **1** are displayed in Fig. 3. The spectrum of the untreated sample looks similar to the spectrum of ferrocenecarboxylic acid reported by Makeieva *et al*.^43^. The bands of the symmetric and assymetric *ν*(COO) modes around 1471 cm^−1^ and 1650 cm^−1^ could be observed as well as the bands of the *γ*(CH) and *δ*(OH) signals around 833 cm^−1^ and 939 cm^−1^ ^44–47^. While the *ν*_*s*_(COO) band lost intensity, a *ω*(CH) band appeared around 800 cm^−1^ and increased as a function of the treatment time^45^. The relative signal intensity of *ω*(CH) band increased in the spectrum of complex **2** with longer treatment times as well, while the *ν*(C=C) band diminished completely^48^. In the spectrum of complex **3**, carbon-related signals such as =C-H around 843 cm^−1^, -CH_3_ around 1320 cm^−1^ and C-OH around 1400 cm^−1^ can be observed^49^. The relative signal intensity of the C=C/C=N bands close to 1618 cm^−1^ increased with increasing treatment time as presented in Fig. 4 ^49^. Although a loss in spectral information due to plasma treatment can be observed for all three iron complexes, the complexes are fairly stable during plasma treatment.

### Stability of iron complexes in the presence of GSH

In the next step the interactions of iron(II) and iron(III) complexes with GSH were investigated. The iron complexes were incubated with GSH in aqueous solution for 1, 3 and 5 min in the ratio 1:1. The mass spectrum with negative ionization mode for complex **1** after 1 min of incubation showed two signals at *m/z* = 229 and at *m/z* = 306, corresponding to the molecular masses of ferrocenecarboxylic acid and the GSH molecule, respectively. The mass spectra for the extended incubation times of 3 and 5 min showed the same ionization signals, confirming the lack of interactions between GSH and the iron(II) complex up to 5 min of incubation. The mass spectrum with positive ionization mode for complex **2** after 1 min of incubation showed two signals of the iron(III) complex at *m/z* = 254 and at *m/z* = 376, corresponding to the [M − L]^+^ and [M + Na]^+^ species. The molecular signal [M + H]^+^ of GSH was found at *m/z* = 308. Moreover, an additional signal at *m/z* = 461 appeared, which illustrate the creation of the iron species consisting of one acetylacetonate ligand and one GSH molecule. Extending the treatment time with plasma had no more impact on the iron and the GSH molecules. The mass spectrum with negative ionization mode for complex **3** after 1 min of incubation showed two signals at *m/z* = 306 and at *m/z* = 611, corresponding to the GSH [M − H]^−^ and hemin [M − H]^−^ molecules. After 3 min of incubation an additional signal appeared at *m/z* = 354, corresponding to the molecular mass of glutathione sulfonic acid GSO_3_H. This signal unveiled the hemin oxidation properties on GSH in aqueous solution. After 5 min of incubation the ratio of all species remained unchanged. The full-scan mass spectra are available in the ESI†.

**Figure 5:**
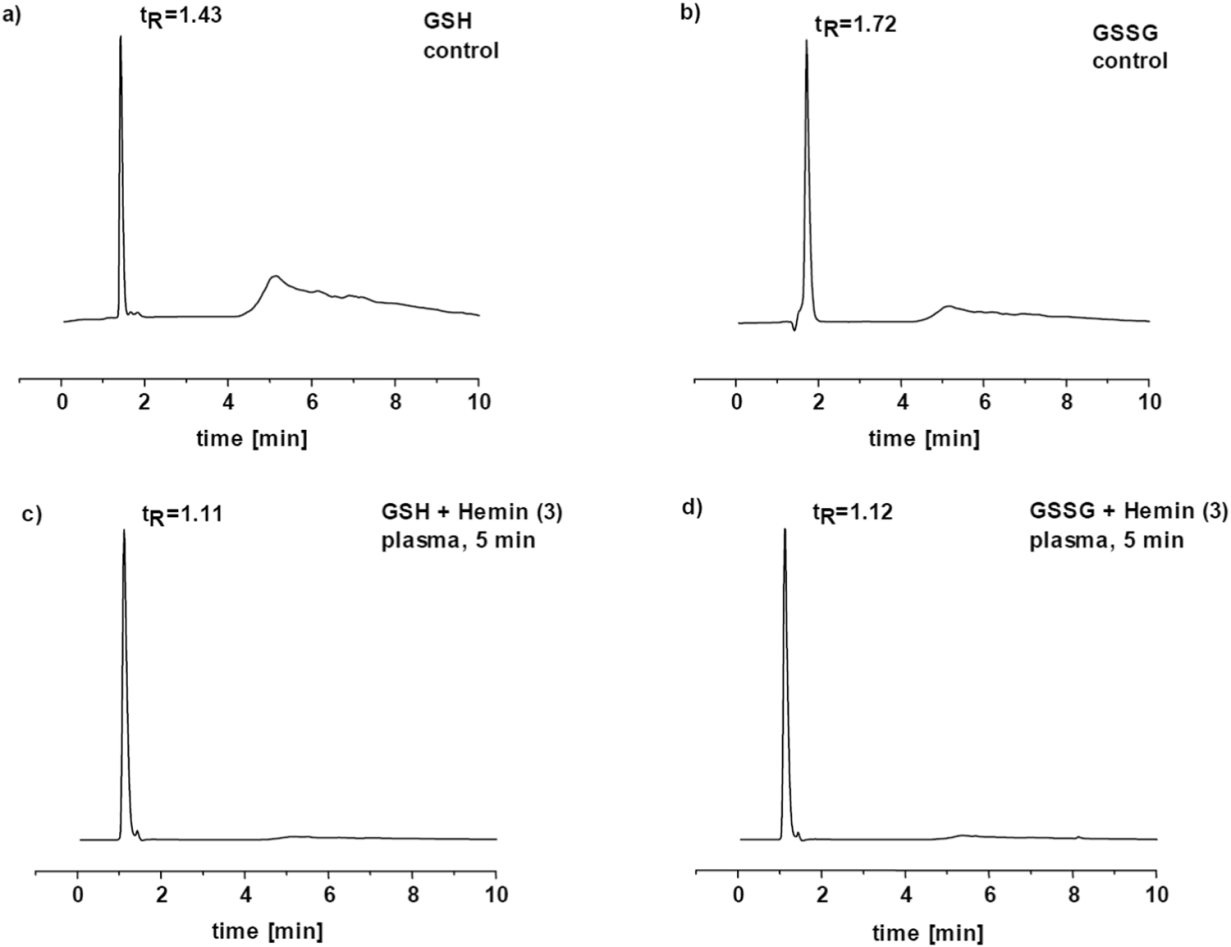
HPLC trace of (**a**) GSH, (**b**) GSSG, (**c**) GSH after plasma treatment in the presence of complex **3**, (**d**) GSSG after plasma treatment in the presence of complex **3**.

To monitor the interactions between GSH molecule and iron complexes also HPLC analysis was involved (Fig. 5). For the interactions of GSH with complex **1** the HPLC chromatogram showed one signal at 1.43 min assigned to the GSH molecule after 1, 3 and 5 min. The HPLC trace for interactions with complex **2** similarly showed a signal of GSH after 1 min of incubation. However, after 3 and 5 min the GSH signal decreased without the presence of different peaks. In the case of incubation with complex **3**, the HPLC trace after 1 min showed one peak corresponding to the GSH molecule. After 3 and 5 min the HPLC profile for complex **3** showed two signals. Of those, the one with retention time at 1.43 min corresponds to GSH, and the new signal at 1.11 min was assigned to GSO_3_H (See the HPLC traces in the ESI†).

### Stability of iron complexes in the presence of GSSG

The negative ionization mode mass spectrum for complex **1** after 1 min of incubation with GSSG showed two signals at *m/z* = 229 and at *m/z* = 611, corresponding to ferrocenecarboxylic acid and the GSSG molecule. The spectra for 3 and 5 min of incubation showed the same ionization pattern with the same ratio. The mass spectrum in positive ionization mode for complex **2** after 1 min of incubation showed three signals, one at *m/z* = 613 corresponding to the GSSG and two signals of an iron complex at *m/z* = 254 and at *m/z* = 376, corresponding to the [M − L]^+^ and [M + Na]^+^ species. After 3 min, the signal at *m/z* = 376 corresponding to [M + Na]^+^ adduct disappeared. Only the iron(III) species without one ligand and the GSSG molecule remained unchanged. The same species were detected after 5 min of incubation. The mass spectrum in positive ionization mode for complex **3** after 1 min of incubation showed two signals at *m/z* = 613 and at *m/z* = 635, corresponding to the [M + H]^+^ and [M + Na]^+^ species of GSSG molecule. The molecular signal [M + H]^+^ of complex **3** was found at *m/z* = 616. The extended incubation time caused no further modification of this iron(III) complex or GSSG molecules.

The HPLC trace for incubation with complex **1** showed one and the same signal at 1.72 min after 1, 3 and 5 min, which was assigned to the GSSG molecule. The chromatogram for plasma treatment of complexes **2** and **3** also showed only one peak of GSSG at 1.72 min, confirming the results from ESI-MS measurements.

### Influence of plasma on GSH in the presence of iron complexes

**Figure 6:**
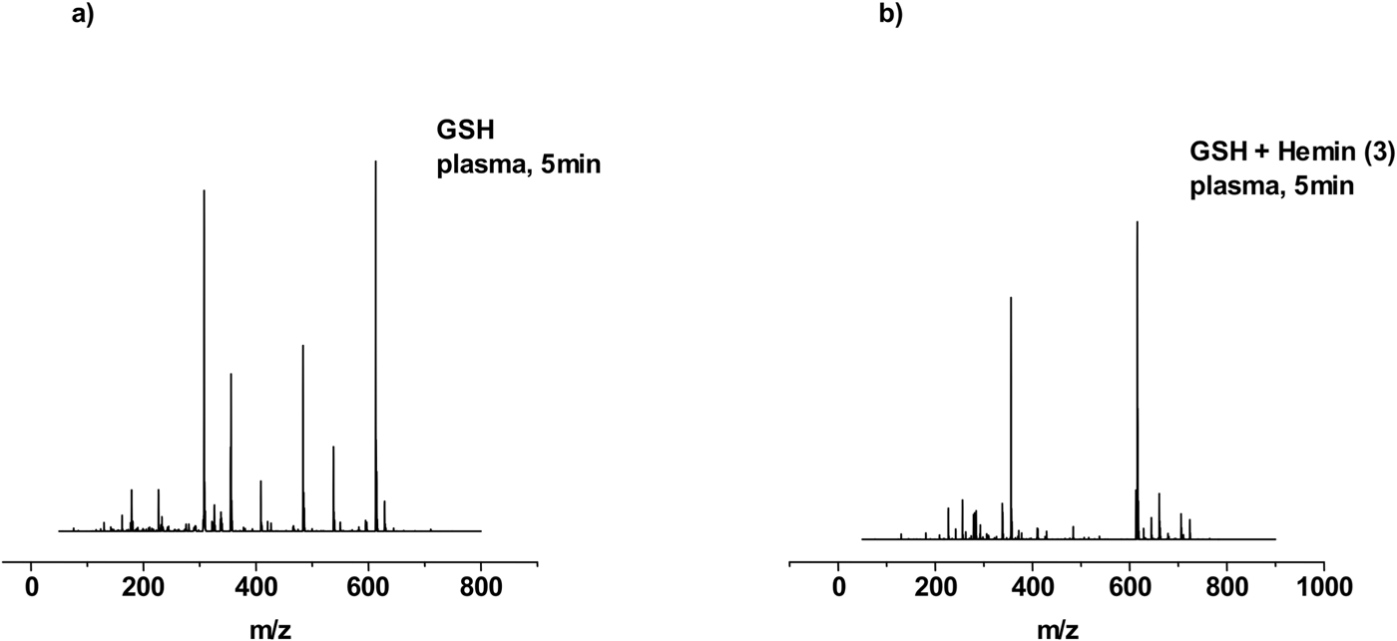
ESI-MS spectra of GSH after plasma treatment for 5 min (**a**) and for GSH after plasma treatment for 5 min in the presence of iron(III) complex. Both spectra were recorded in positive mode.

As the last step the impact of plasma treatment on GSH and GSSG in the presence of iron(II) and iron(III) complexes was investigated. Measurements of the sample after treatment of GSH in the presence of complex **1** with the DBD for 1 min showed the signal at *m/z* = 229 corresponding to the [M − H]^−^ moiety of ferrocenecarboxylic acid (**1**). No molecular ion peak [M + H]^+^ corresponding to GSH was found. However, the spectrum exhibited an additional signal at *m/z* = 354 with high intensity, which corresponds to glutathione sulfonic acid GSO_3_H. The samples after 3 and 5 min of treatment displayed the presence of the same species. For complex **2**, the ESI-MS spectrum after 1 min revealed four signals at *m/z* = 256, 282, 356 and 563. The two most intensive signals are found at *m/z* = 256 and 282, which are assigned to the iron(III) complex, gradually loosing O-donor ligands. The signal at *m/z* = 354 with moderate intensity can be assigned to glutathione sulfonic acid GSO_3_H. The signal at *m/z* = 563 corresponds to the iron(III) complex with one acetylacetonate ligand substituted by a GSH molecule. After 3 min, signals at *m/z* = 256 and 282 remained with the same ratio of intensity as well as the signal at *m/z* = 563. Interestingly, the signal at *m/z* = 354 assigned to glutathione sulfonic acid GSO_3_H was not observed. The spectrum after 5 min presented the same observation like the spectrum after 3 min of treatment. The mass spectrum of complex **3** for 1 min showed two major signals at *m/z* = 356 and at *m/z* = 616, corresponding to the glutathione sulfonic acid GSO_3_H and hemin molecules, respectively. No signal corresponding to the GSH molecule was observed. Identical product-ion spectra were recorded after 3 and 5 min of plasma treatment (Fig. 6).

In Fig. 8, the FTIR-spectra of untreated and plasma-treated GSH in combination with complex **1** are depicted. In addition to the bands formed during plasma treatment of complex **1** only, two additional bands appeared. The band at 1040 cm^−1^ can be assigned to the S=O stretch vibration and the band around 1739 cm^−1^ indicates the formation of *ν*(C=O)^45,49,50^. The same observation was made regarding GSH in the presence of complex **2** as well as complex **3**. The relative signal intensity of the *ω*(CH) band at 800 cm^−1^ increased as a function of the treatment time in the presence of complex **2**. In the spectra of GSH combined with complex **3**, a diminishing amide band can be observed around 1560 cm^−1^.

**Figure 7:**
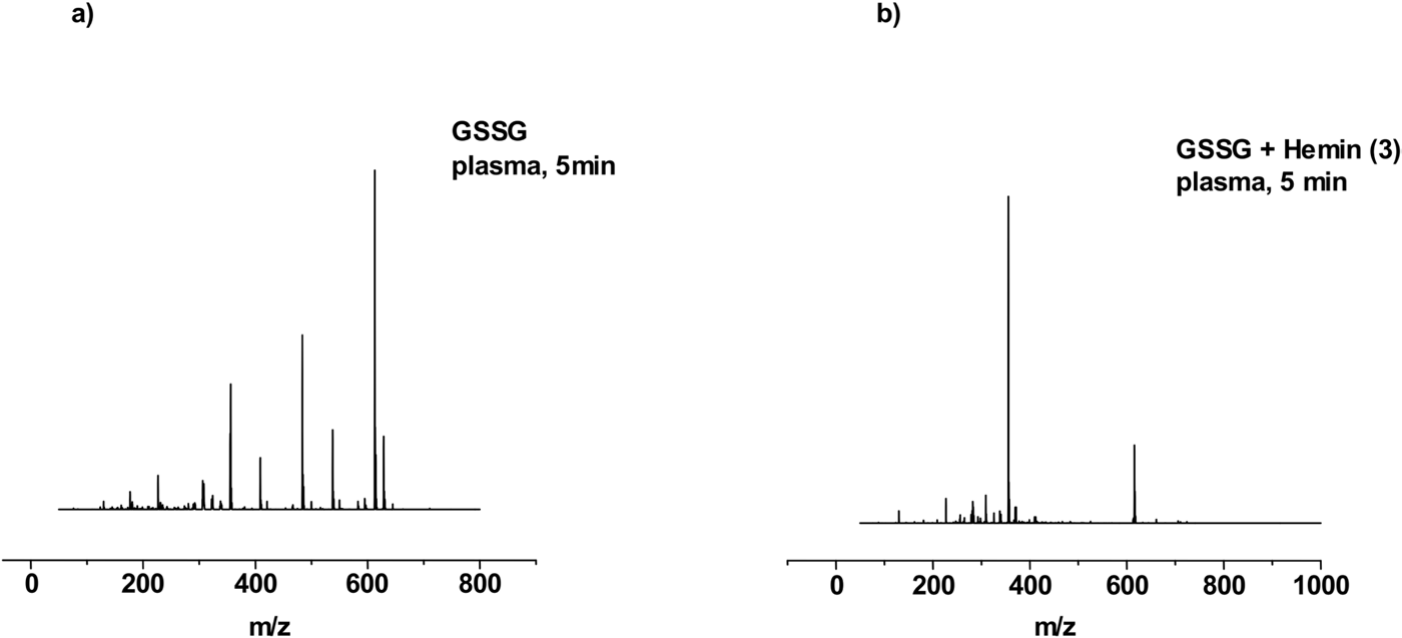
ESI-MS spectra of GSSG after plasma treatment for 5 min (**a**) and for GSSG after plasma treatment for 5 min in the presence of iron(III) complex. Both spectra were recorded in positive mode.

The samples after plasma treatment of GSH in the presence of iron complexes were analyzed also by HPLC. The HPLC trace for GSH in the presence of complex **1** after 1, 3 and 5 min of plasma treatment showed one signal at 1.11 min, which is again assigned to the GSO_3_H molecule. The chromatogram of GSH in the presence of complex **2** showed the same peak after 1 min. After 3 and 5 min a peak at 1.11 min disappeared. The HPLC profile of GSH in the presence of complex **3** showed also only one signal after 1, 3 and 5 min, corresponding to the GSO_3_H molecule (Fig. 5).

### Influence of plasma on GSSG in the presence of iron complexes

In the mass spectra of complex **1** after 1 min of treatment the molecular ion peak [M − H]^−^ from this unchanged iron(II) complex was found at *m/z* = 229. The spectrum exhibited only one more species at *m/z* = 354 assigned to [M − H]^−^ glutathione sulfonic acid GSO_3_H. After 3 min of plasma treatment the signal at *m/z* = 229 was not anymore recorded giving glutathione sulfonic acid GSO_3_H as the only product. The spectrum for plasma-treated GSSG for 5 min in the presence of ferrocene carboxylic acid presented also only a signal at *m/z* = 354 assigned to GSO_3_H. The spectrum of complex **2** after 1 min of treatment, despite the signal of the iron(III) complex, exhibited two additional signals at *m/z* = 356 and at *m/z* = 613, assigned to GSO_3_H and GSSG, respectively. Extending the treatment time to 3 and 5 min had no impact on GSSG modification. For complex **3**, after 1 min of plasma treatment, the most intensive peak in the mass spectra was found at *m/z* = 356, which corresponds to GSO_3_H species. Moreover, the signal at *m/z* = 616 was present with moderate intensity and is assigned to the hemin molecule. No signal from GSSG molecule was observed during measurements. The mass spectra for 3 and 5 min also showed only these two species with the same ratio (Fig. 7).

**Figure 8:**
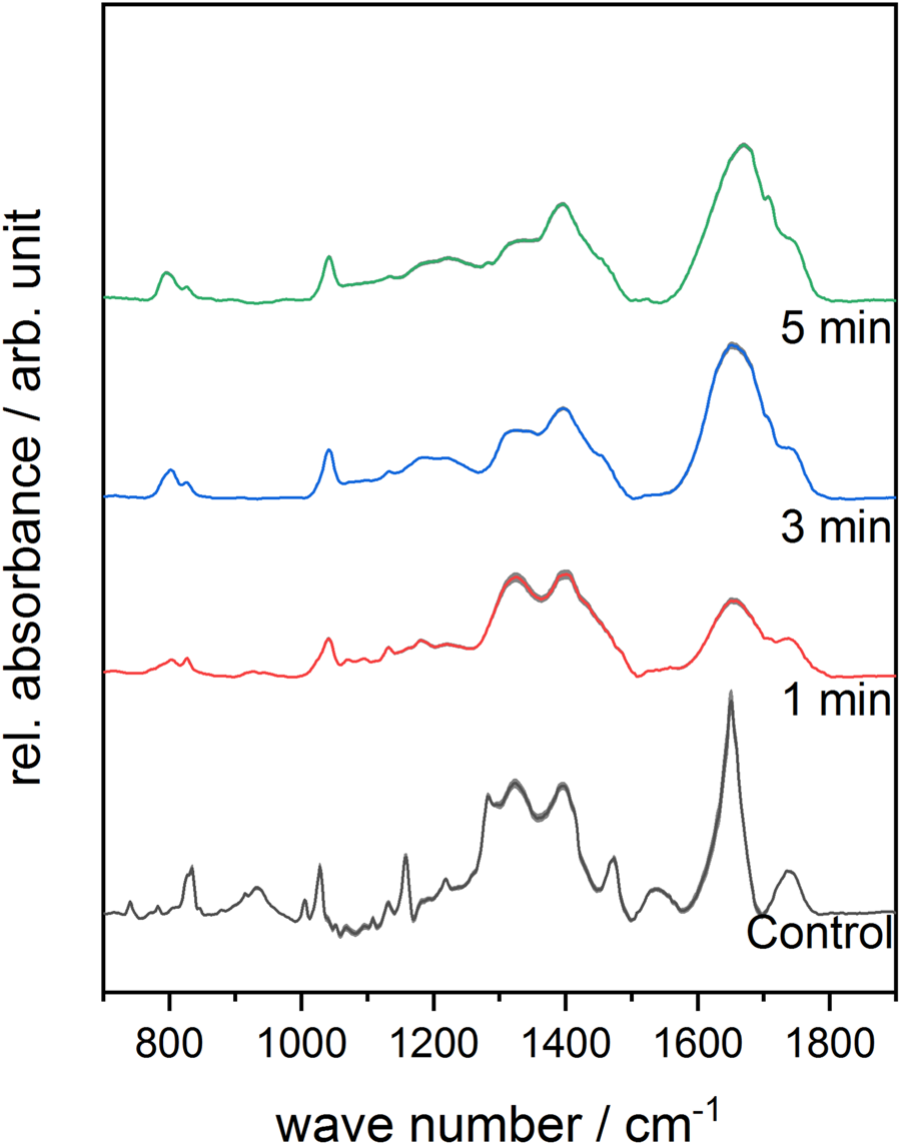
Mean FTIR-spectra of plasma-treated GSH in the presence of Complex **1** within the range of 700–1900 cm^−1^ as a function of different treatment times. Standard deviation of the mean is shown as grey area at each graph.

**Figure 9:**
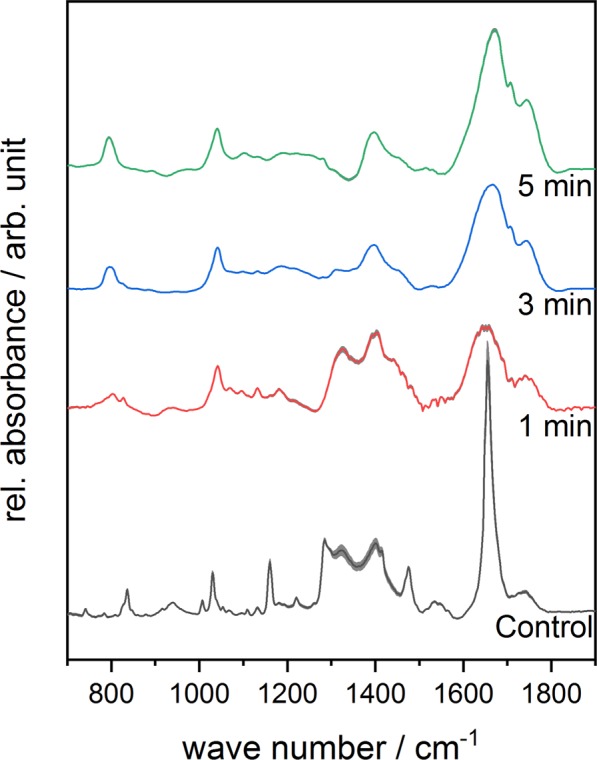
Mean FTIR-spectra of plasma-treated GSSG in the presence of Complex **1** within the range of 700–1900 cm^−1^ as a function of different treatment times. Standard deviation of the mean is shown as grey area at each graph.

In the FTIR-spectra of GSSG in presence of complex **1** (Fig. 9), complex **2** and complex **3**, the band at 1040 cm^−1^, which is assigned to the S=O stretching vibration, was formed during treatment. This band was already observed in the spectra of GSH in combination with the complexes. Moreover, the relative signal intensity of the *ν*(C=O) bond related to the carboxyl group increased with longer treatment times.

In the chromatogram of the plasma-treated GSSG in the presence of complex **1**, only one peak was observed at 1.12 min, which corresponded to the GSO_3_H species. In the presence of complex **2** the HPLC trace after 1 and 3 min showed two peaks at 1.12 min and 1.72 min, corresponding to GSO_3_H and GSSG, respectively. After 5 min only one peak corresponding to GSSG was observed. The HPLC chromatograms after plasma treatment in the presence of complex **3** presented one signal at 1.12 min indicating the presence of GSO_3_H as oxidation product, regardless of the treatment time (Fig. 5).

## Discussion

**Figure 10:**
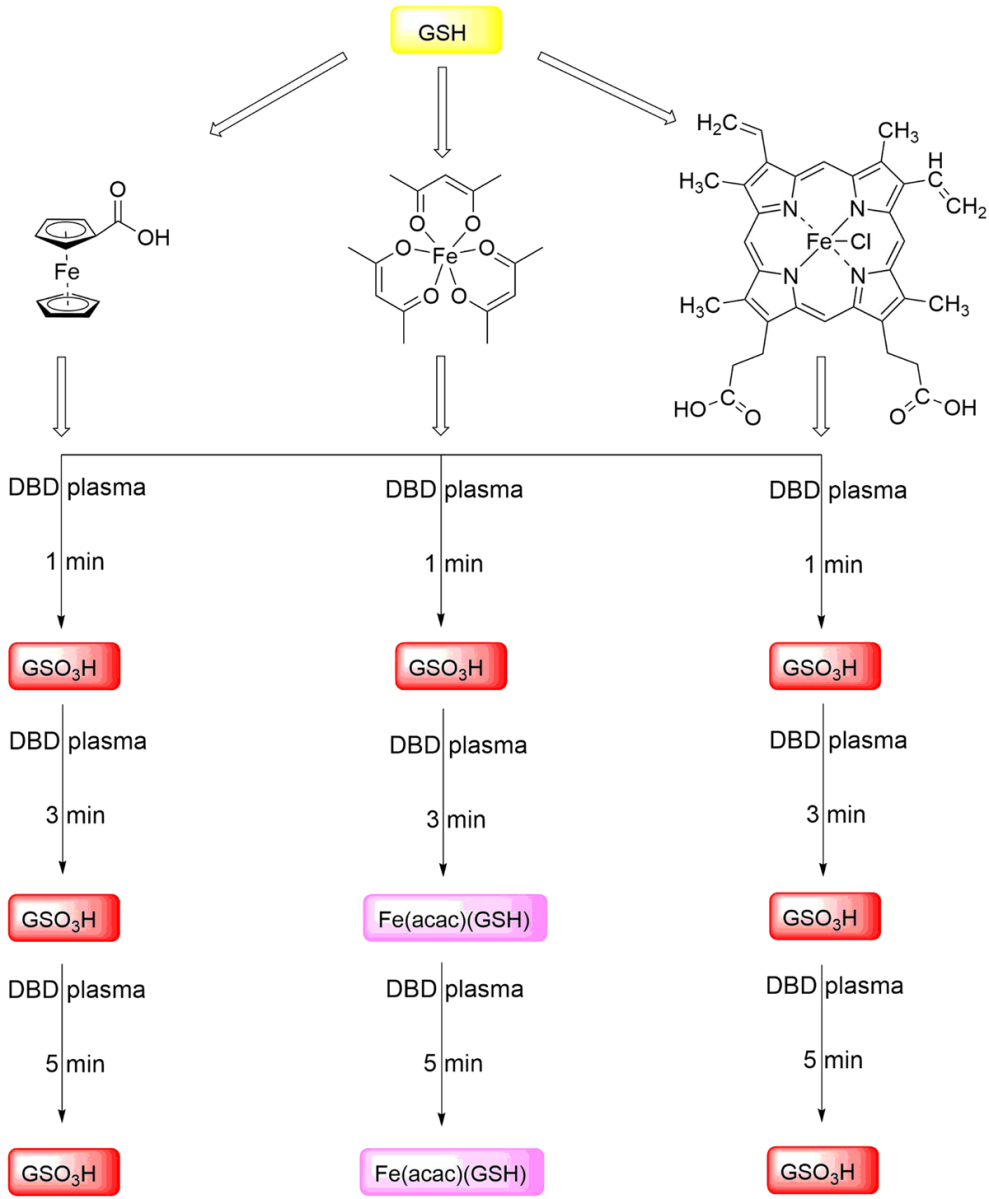
Chemical modifications of GSH after different times of plasma treatment in the presence of iron complexes.

**Figure 11:**
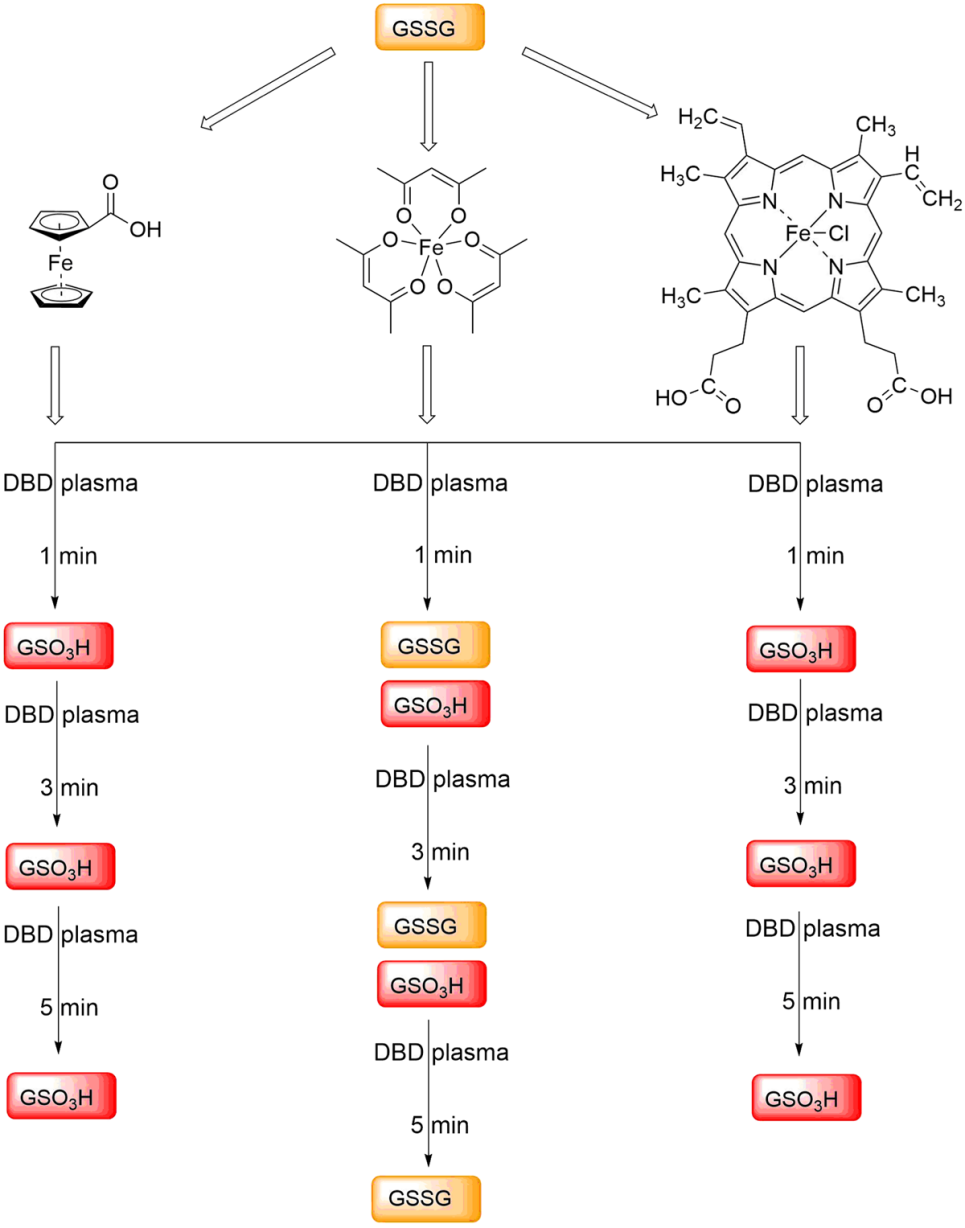
Chemical modifications of GSSG after different times of plasma treatment in the presence of iron complexes.

According to our previous findings, plasma treatment caused a number of chemical modifications on GSH and GSSG in solution, giving a large number of (partly unidentified) S- and N-modified glutathione species as intermediates and final products (Fig. 1). That tendency changes drastically in the presence of iron(II) and iron(III) complexes (Figs. 10 and 11). Our findings show that plasma treatment causes clean oxidation of GSH as well as GSSG to one product only, namely GSO_3_H. These data provide important insights into the effects of iron complexes on chemical modifications of biomolecules by non-thermal plasma. Our results highlight the role of such studies for future clinical applications, because of the high level of iron species in biological organisms^51,52^.

In order to identify the chemical modifications of GSH and GSSG induced by plasma treatment in the presence of iron complexes, we investigated the samples by Mass Spectrometry (ESI-MS), High Performance Liquid Chromatography (HPLC) and IR spectroscopy. We monitored changes depending on the treatment time for several spectral features. The ESI-MS investigations were conducted in both negative and positive scanning mode enabling detection of both positively and negatively charged GSH and GSSG species, as well as (modified) iron(II) and iron(III) complexes. The stability experiments on complexes **1**, **2** and **3** were carried out in aqueous solution for 1, 3, 5 and 20 min. All complexes showed unchanged, stable molecule ions during the exposure time, which made them suitable candidates for our plasma exposure experiments. Next, the plasma influence on all three iron complexes was monitored by ESI-MS and IR. Ferrocene carboxylic acid (**1**) and the hemin (**3**) complex were not affected by plasma treatment within 1 and 3 min. After 5 min only minor changes compared to untreated samples could be observed, showing the loss of H_2_O molecules. On the other hand, treatment of the iron(III) acetylacetonate complex **2** with the DBD caused the loss of one acetylacetonate moiety.

Before elucidation of the impact of plasma treatment on GSH and GSSG in the presence of iron complexes, the direct interactions between GSH/GSSG and the iron complexes were investigated. Each iron complex was incubated in water with GSH or GSSG in the ratio 1:1 for 1, 3 and 5 min. Complex **1** showed no interaction either with GSH or with GSSG, and the same was true for complex **3** with GSSG. Also extending the incubation times had no influence. After the incubation of hemin (**3**) with GSH, MS measurements and HPLC analysis revealed oxidation of GSH to glutathione sulfonic acid GSO_3_H, but still GSH was observed. In the case of the iron(III) complex **2**, substitution of an acetylacetonate ligand by GSH already after 1 min was observed. After 5 min all GSH molecules were converted to GS-iron(III) species. On the other hand, incubation with GSSG caused no interactions.

Finally, the impact of plasma on GSH and GSSG in the presence of iron complexes was investigated. For the plasma treatment of GSH in the presence of iron(II) complex (**1**) and iron(III) complex (**3**), GSO_3_H was observed as the only species, indicating complete oxidation of the thiol moiety of GSH. Extending the treatment times revealed the same species, indicating that GSO_3_H is the final and the only oxidation product. The same observations were made for treatment of GSSG in the presence of iron(II) complex (**1**) and iron(III) complex (**3**), both resulting in the immediate loss of GSSG signal intensity. In the case of the more labile complex **2**, GSH was preliminary converted to GSO_3_H, which nonetheless disappeared with time and new iron(III) species were formed as the final product. In the presence of GSSG, GSO_3_H could be observed as an increasing signal. However, after 3 and 5 min of plasma treatment, the signal assigned to GSSG increased again along with decrease of the previously formed GSO_3_H. All ESI-MS and HPLC investigations described above were corroborated by the changes in the FTIR spectra. These indicated loss of thiol signals and increasing intensity of S=O bands, thereby confirming an oxidation of the free thiol group of GSH by DBD treatment in the presence of iron complexes. All taken together, our findings suggest that the presence of iron(II) and iron(III) complexes during plasma treatment causes rather selectively chemical modifications on GSH and GSSG by converting them selectively into GSO_3_H. Surprisingly, no nitrosoglutathione or derivatives thereof were observed during plasma treatment in the presence of iron complexes. Hence, based on our results, we suggest that ROS play the leading role in the modification of GSH and GSSG molecules in the presence of iron complexes. Similar conclusions were made in the case of N-acetyl cysteine^53^. For further studies we suggest to involve complexes **1** and **3**, possessing strongly bound ligands, as suitable model complexes both biologically relevant oxidation states +II and +III, with good solubility in water and biological media. Complex **2**, on the other hand, does not appear as a good model iron complex, because of the relative ease of ligand loss and interaction with GSH, even before plasma treatment.

## Materials and Methods

### Experimental setup

The experiments in this study were carried out with a dielectric barrier discharge, which consists of a copper electrode covered with aluminium oxide (Al_2_O_3_) with a thickness of 1 mm. The electrode has a diameter of 10 mm and the distance between the driven electrode and the sample was kept constant at 1 mm. The samples were placed on a grounded aluminium plate and ambient air was used as the process gas. The temperature in the lab was adjusted to 20 °C and the relative humidity varied between 40% and 50% during the period in which the experiments were carried out. The experimental setup is described in more detail in Kogelheide *et al*. and a scheme of the dielectric barrier discharge can be found in the ESI†^54^. The electrode was driven with a pulsed power supply^55^. For the experiments in this study the repetition frequency was set to 300 Hz and the amplitude of the HV pulse to 24 kV_*pp*_. The dielectric barrier discharge has been characterised regarding several plasma parameters as well as reactive species. In Kogelheide *et al*., the electron density distribution in the discharge is described in detail^55^. The radial profiles of the plasma produced oxygen species, atomic oxygen (O) and ozone (O_3_), within the plasma volume of the former used plasma source are determined using two-photon laser-induced fluorescence spectroscopy (TALIF) and optical absorption spectroscopy (OAS) in Baldus *et al*. Furthermore, a model of the afterglow chemistry is described in this paper to obtain insight into the dynamics of the considered reactive oxygen species^56^.

### Materials

All reagents and chemicals were purchased from commercial sources and used without further purification. Ferrocenecarboxylic acid was purchased from Abcr. Iron(III) acetylacetonate (Fe(acac)_3_), chloro(protoporphyrinato)iron(III) (hemin), L-Glutathione reduced (GSH), L-Glutathione oxidized (GSSG) and N,N-Diisopropylethylamine (DIEA) were purchased from Sigma-Aldrich.

### Glutathione sample preparation

L-glutathione in oxidised (GSSG) and reduced (GSH) state were dissolved in distilled water with a concentration of 4 mg/ml. Ferrocenecarboxylic acid, Iron(III) acetylacetonate (Fe(acac)_3_) and chloro(protoporphyrinato)iron(III) (hemin) were dissolved in distilled water with a concentration of 4 mg/ml with 2 eq. of DIEA. 10 *μ*l were placed on cleaned silicon wafers (Siltronic AG) and treated with the DBD for 1, 3 and 5 min. After treatment, samples were filled into reagent tubes and evaporated liquid replenished with A. dest to the concentration of 1 mg/mL for the analysis via mass spectrometry and HPLC. The samples for the FTIR spectroscopy were dried by desiccation after the plasma treatment. As controls, another sample was prepared equally, omitting the plasma treatment. Control samples were placed in ambient conditions as the sample treated for the longest time.

### Mass spectrometry

Electron spray ionization (ESI) mass spectra were obtained on an Esquire 6000 mass spectrometer (Bruker). Full mass spectra of the investigated ferrocenecarboxylic acid, iron(III) acetylacetonate (Fe(acac)_3_), chloro(protoporphyrinato)iron(III) (hemin), GSH and GSSG were acquired in both negative-ion and positive-ion mode with the spectrometer equipped with an ion-trap analyser. Three samples of 10 *μ*l treated for the same time were pooled and diluted tenfold with acetonitrile for 300 *μ*l with a final concentration of 1 mg/ml. Instrumental parameters were tuned for each sample. The capillary voltage was set in a range of −22 to 25 V, the spray voltage was between 3.00 and 4.50 kV, and a capillary temperature of 180 °C was employed. The mass scan range was from *m/z* 50 to 2000 amu, for 20 s scan time. Spectra were acquired using a direct infusion setup with a flow rate of 5 *μ*l/min with a cone voltage of 20 kV. To determine occurring in-source fragments, which increase the sample complexity without yielding significant additional information, MS/MS spectra were acquired using the same conditions with a collision energy ramp between 2.00 and 4.00 eV. Spectra were deconvoluted and a background of ten times noise (500 counts in positive and 5 counts in negative mode) was subtracted before peak annotation. All experiments were performed in triplicates.

### FTIR spectroscopy

A Bruker Vertex FTIR-micro spectrometer was used for the analysis of the samples. FTIR spectra were recorded from 750 cm^−1^ to 4000 cm^−1^ with a spectral resolution of 4 cm^−1^. For the FTIR spectroscopy of the investigated Ferrocenecarboxylic acid, iron(III) acetylacetonate (Fe(acac)_3_), chloro(protoporphyrinato)iron(III) (hemin), GSH and GSSG 12 spectra were recorded at different positions of each sample with each spectrum representing 32 accumulated spectra. Background spectra were obtained for every samples due to the ambient measurement conditions to compensate water and carbon dioxide content in air. All recorded transmission spectra, *T*, were converted into absorption spectra, *A*:

1 $$A=\,\log \,(\frac{1}{T}).$$

Absorption spectra were baseline corrected afterwards and normalization of the data was carried out applying the Euclidean norm:

2 $${a}_{k}^{norm}=\frac{{a}_{k}}{\sqrt{{\sum }_{k=1}^{n}{({a}_{i})}^{2}}}.$$

Every data point of each spectrum *a*_*k*_ of wavenumber *k* is normalized to the square root of the sum of every spectrum data point. All experiments were performed in triplicates.

### HPLC

An HPLC Knauer system with a quaternary pump and a UV-DAD detector equipped with a Nucleodur® C4 ec column (125 mm 4 mm, internal diameter 5 *μ*m, Macherey-Nagel), was used. HPLC was performed by using two buffer systems (buffer A: H_2_O/MeCN/TFA, 95:5:0.1, v/v/v; buffer B: MeCN/H_2_O/TFA, 95:5:0.1, v/v/v) as the mobile phase. Chromatography was performed with a linear gradient conditions of buffer B (100% in 10 min) from 100% buffer A with a total run time of 50 min. The flow rate of the mobile phase was 1.0 ml/min. 10 *μ*l of the sample was injected. The column was purged with the mobile phase for 2 min, followed by equilibration for 15 min, and then 15 min were required for sample analysis. Spectral data were collected at detection wavelengths of 214 nm and 254 nm, and finally the collected data were plotted.

## Supplementary information

Supplementary Information
