# Supplementary material for: Study on Chemical Modifications of Glutathione by Cold Atmospheric Pressure Plasma (Cap) Operated in Air in the Presence of Fe(II) and Fe(III) Complexes
Source: Sci Rep. 2019 Dec 2;9:18024. doi: 10.1038/s41598-019-53538-y (PMC6888970; doi:10.1038/s41598-019-53538-y)
Supplement: Supplementary file 1 — Supplementary Information [file 41598_2019_53538_MOESM1_ESM.pdf]

Electronic Supporting Information

for

STUDY ON CHEMICAL MODIFICATIONS OF GLUTATHIONE BY COLD ATMOSPHERIC PRESSURE PLASMA (CAP) OPERATED IN AIR IN THE PRESENCE OF Fe(II) AND Fe(III) COMPLEXES

by

Dariusz Śmiłowicz[a], Friederike Kogelheide[b], Katharina Stapelmann[c], Peter Awakowicz[b], Nils Metzler-Nolte[*, a]

[a] Chair of Inorganic Chemistry I – Bioinorganic Chemistry, Faculty of Chemistry and Biochemistry, Ruhr-University Bochum, Bochum, Germany

[b]Institute for Electrical Engineering and Plasma Technology, Ruhr University Bochum, 44780, Bochum, Germany

[c] Department of Nuclear Engineering, North Carolina State University, Raleigh, North Carolina 27695, USA

Contents

1. Characterisation of iron(II) and iron(III) complexes p.S2-S5
2. Results of stability experiments p.S5-S11
3. Influence of plasma on iron complexes p.S11-S17
4. Incubation of iron complexes with GSH p.S17-S21
5. Incubation of iron complexes with GSSG p.S22-S26
6. Influence of plasma on GSH in the presence of iron complexes p.S26-S32
7. Influence of plasma on GSSG in the presence of iron complexes p.S32-S44
8. Scheme of the plasma source p.S44

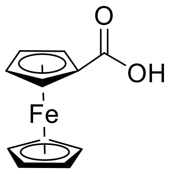

Chemical Formula: $C_{11}H_{10}FeO_2$
Exact Mass: 230,00
Molecular Weight: 230,04

Figure S1. Structure of complex **(1)**.

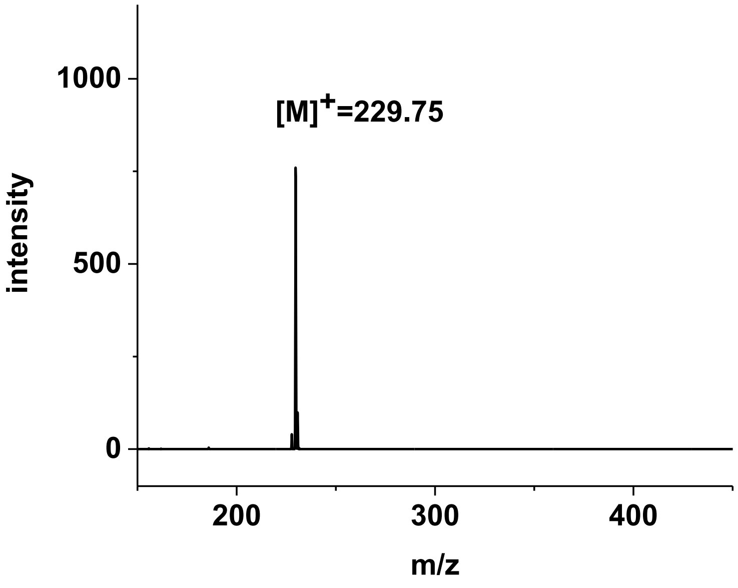

Figure S2. ESI-MS spectrum of complex (**1**).

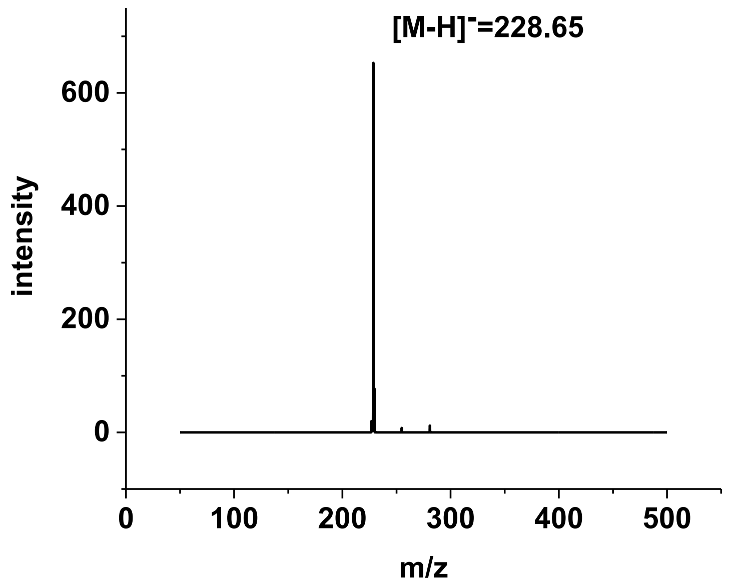

Figure S3. ESI-MS spectrum of complex (**1**).

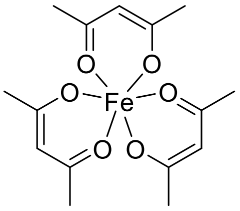

Chemical Formula: $C_{15}H_{21}FeO_6$
Exact Mass: 353,07
Molecular Weight: 353,17

Figure S4. Structure of complex **(2)**.

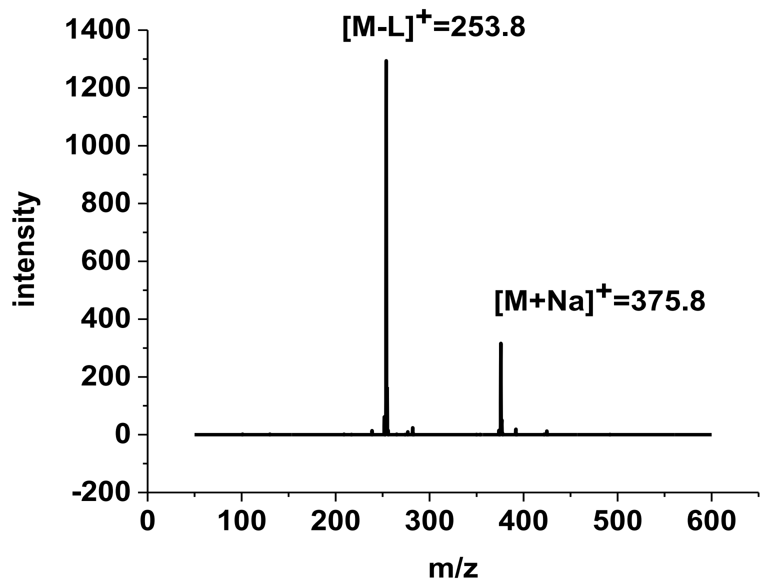

Figure S5. ESI-MS spectrum of complex (**2**).

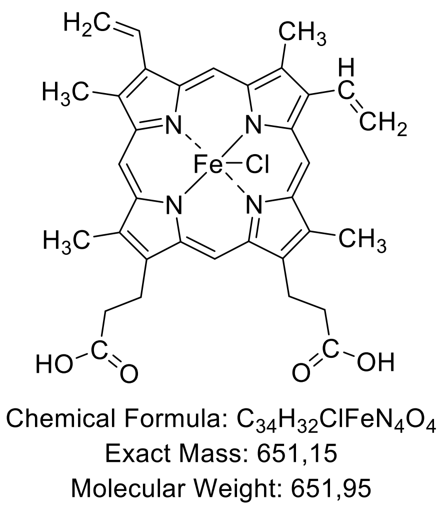

Figure S6. Structure of complex **(3)**.

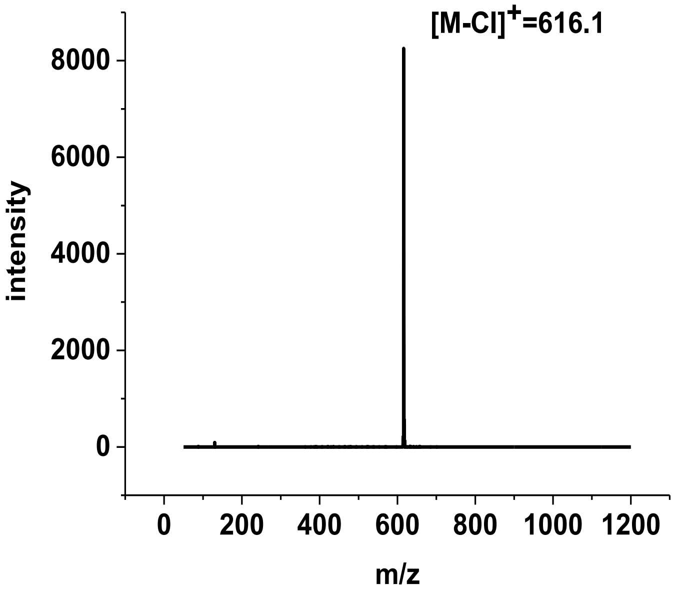

Figure S7. ESI-MS spectrum of complex (**3**).

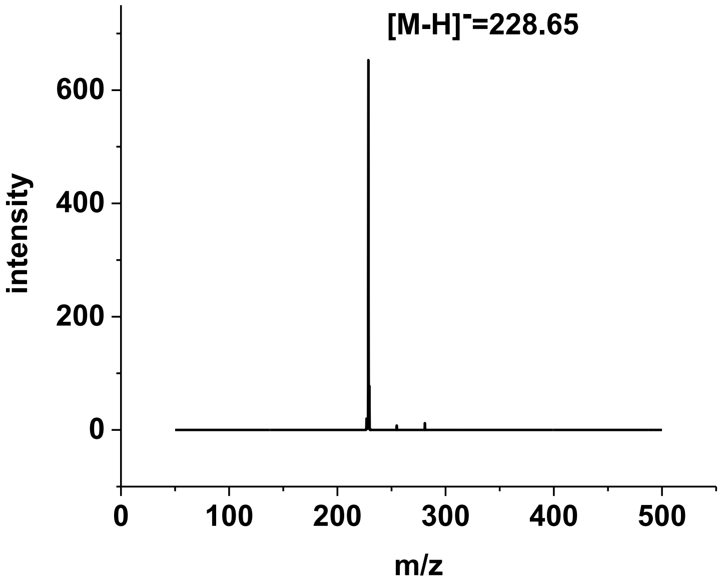

Figure S8. ESI-MS spectrum of complex (**1**) after 1 min of stability experiments.

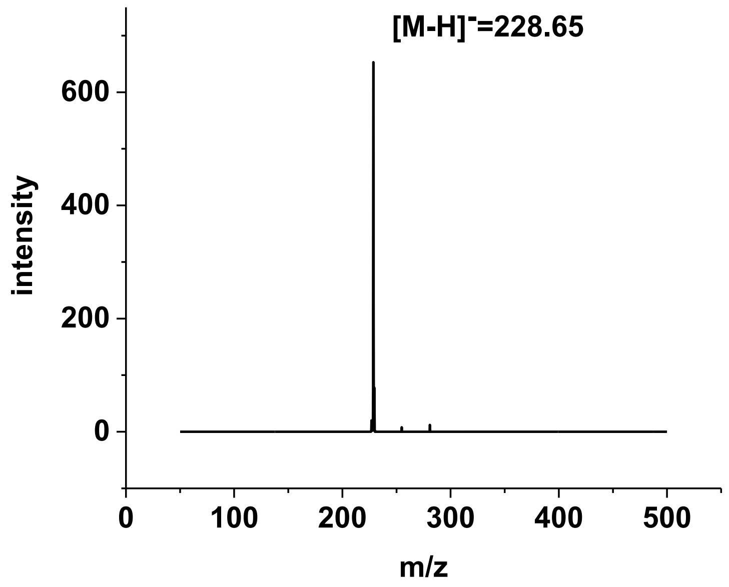

Figure S9. ESI-MS spectrum of complex (**1**) after 3 min of stability experiments.

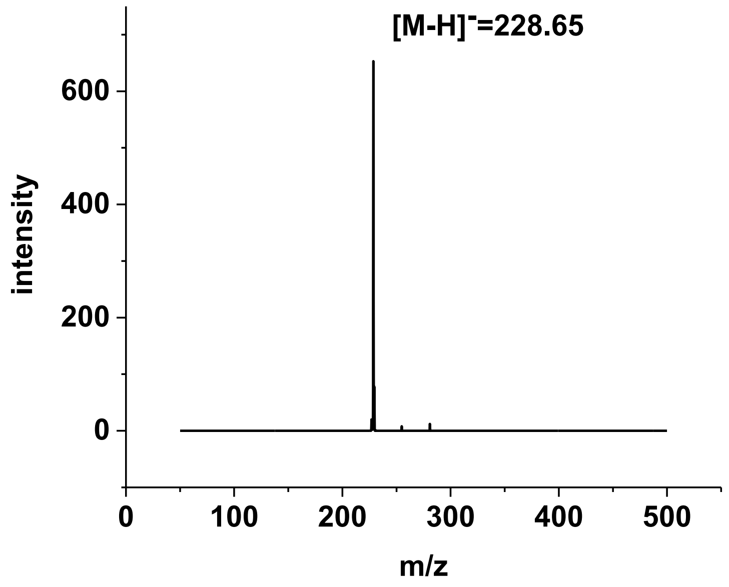

Figure S10. ESI-MS spectrum of complex (**1**) after 5 min of stability experiments.

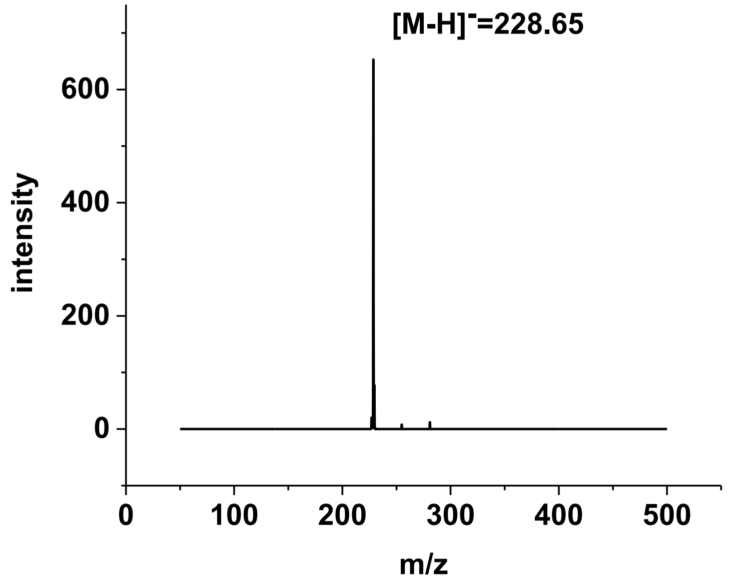

Figure S11. ESI-MS spectrum of complex (**1**) after 20 min of stability experiments.

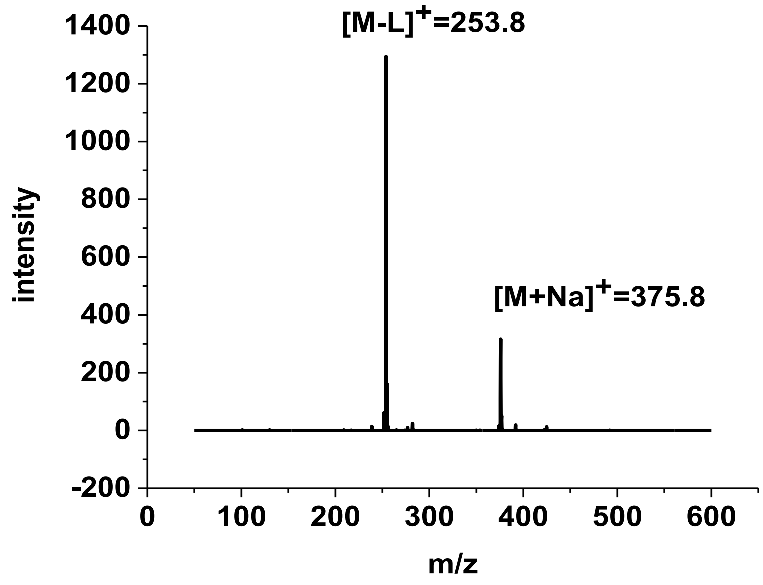

Figure S12. ESI-MS spectrum of complex (**2**) after 1 min of stability experiments.

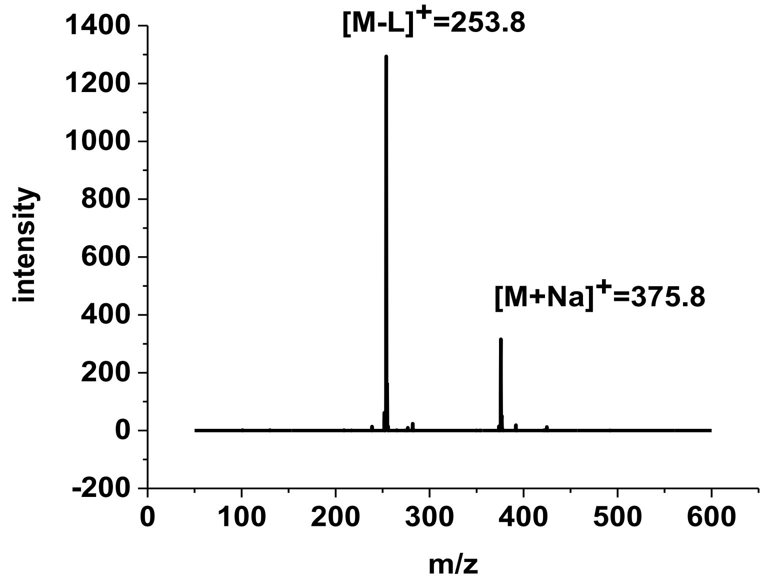

Figure S13. ESI-MS spectrum of complex (**2**) after 3 min of stability experiments.

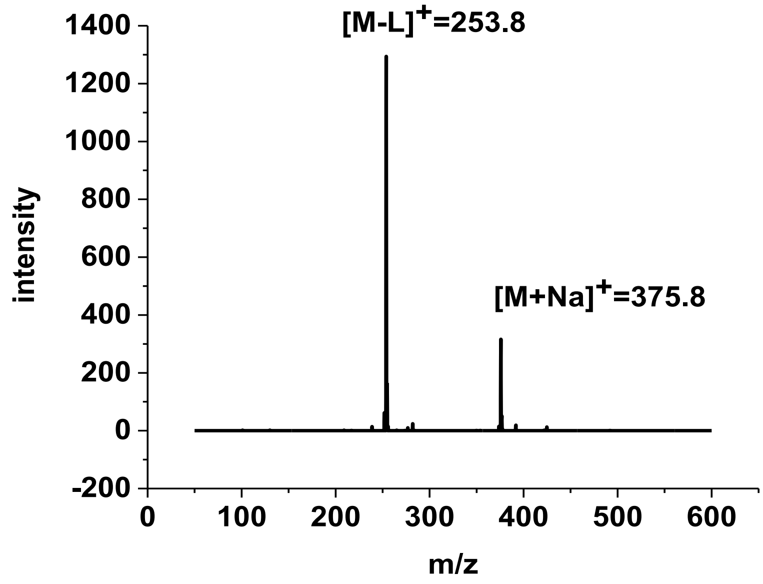

Figure S14. ESI-MS spectrum of complex (**2**) after 5 min of stability experiments.

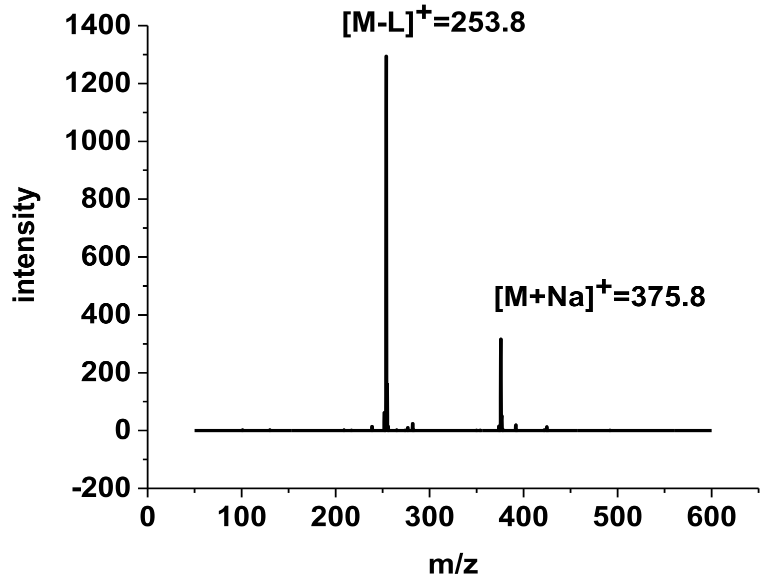

Figure S15. ESI-MS spectrum of complex (**2**) after 20 min of stability experiments.

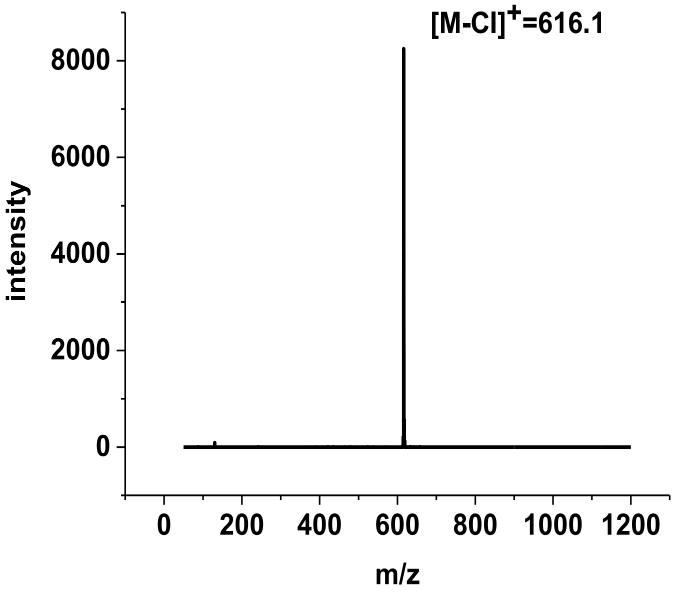

Figure S16. ESI-MS spectrum of complex (**3**) after 1 min of stability experiments.

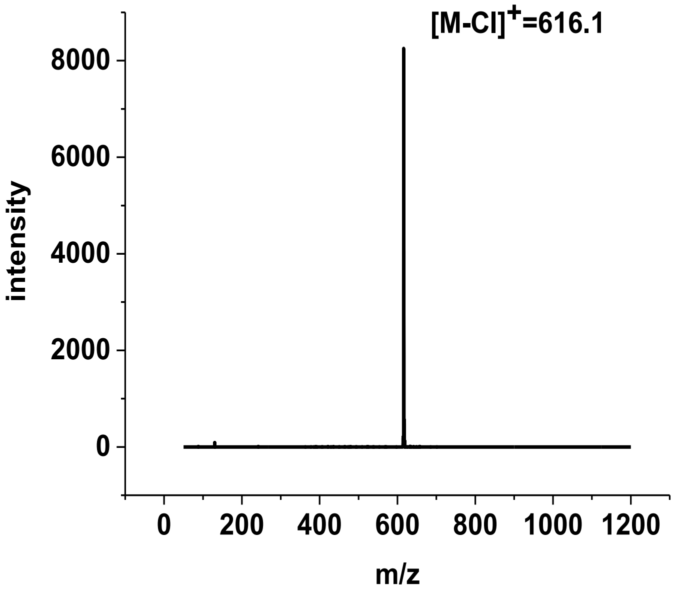

Figure S17. ESI-MS spectrum of complex (**3**) after 3 min of stability experiments.

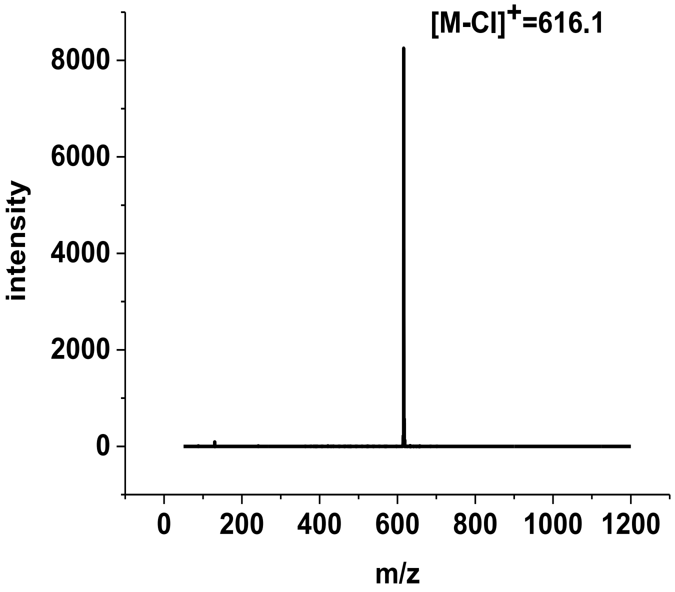

Figure S18. ESI-MS spectrum of complex (**3**) after 5 min of stability experiments.

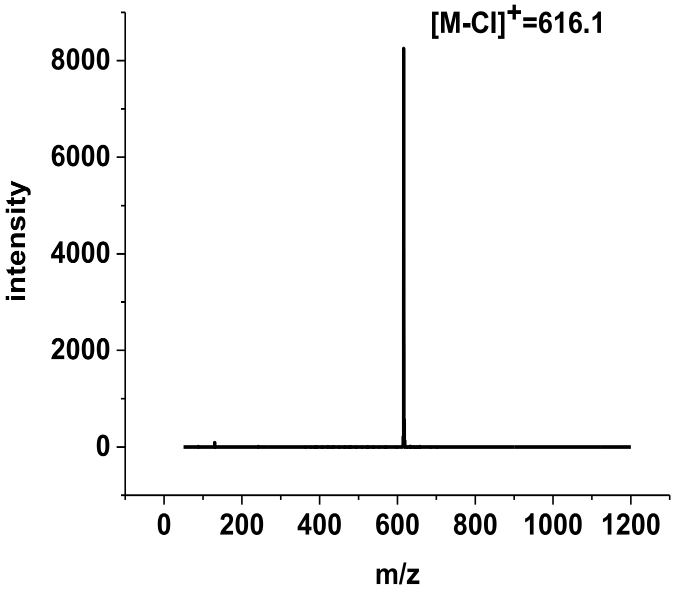

Figure S19. ESI-MS spectrum of complex (**3**) after 20 min of stability experiments.

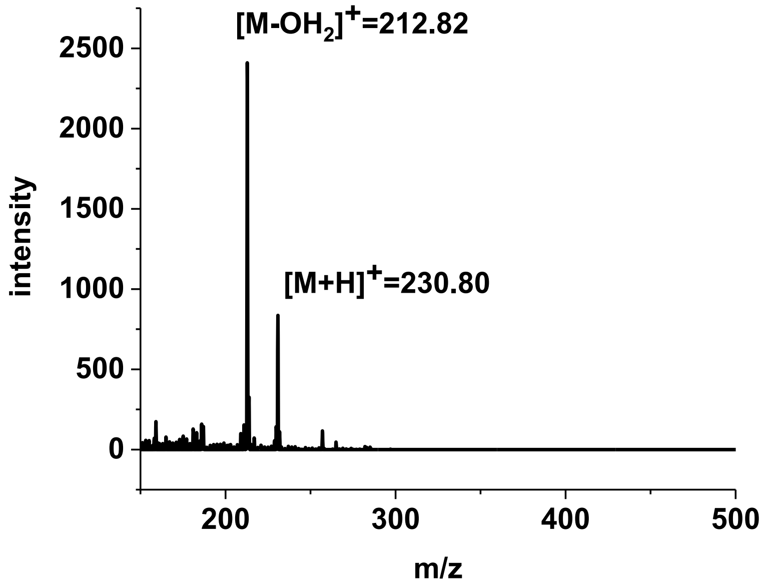

Figure S20. ESI-MS spectrum of complex (**1**) after 1 min of plasma treatment.

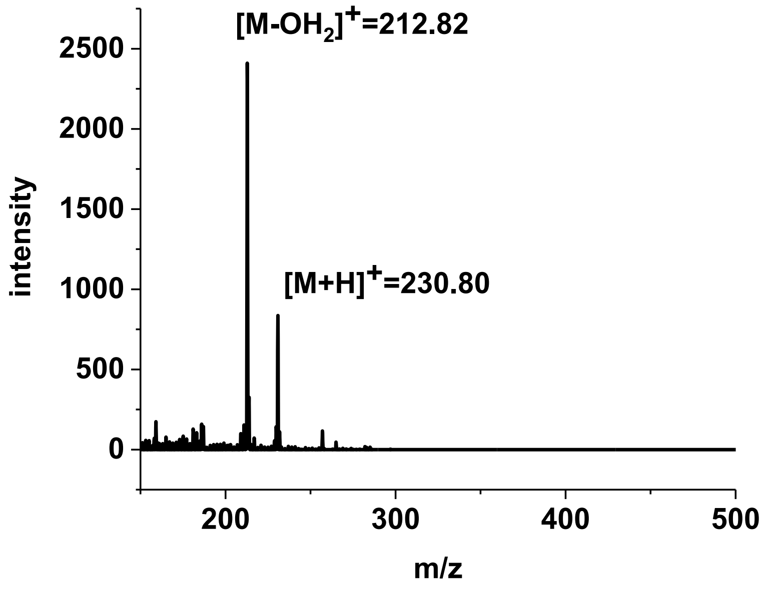

Figure S21. ESI-MS spectrum of complex (**1**) after 3 min of plasma treatment.

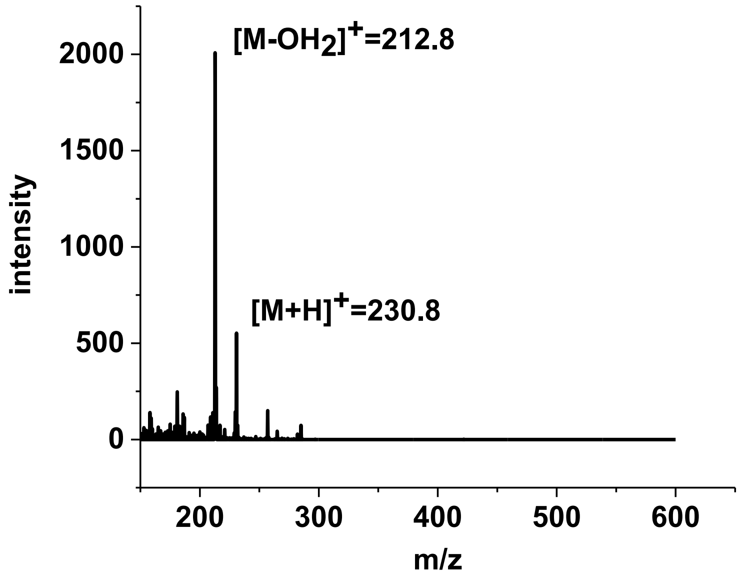

Figure S22. ESI-MS spectrum of complex (**1**) after 5 min of plasma treatment.

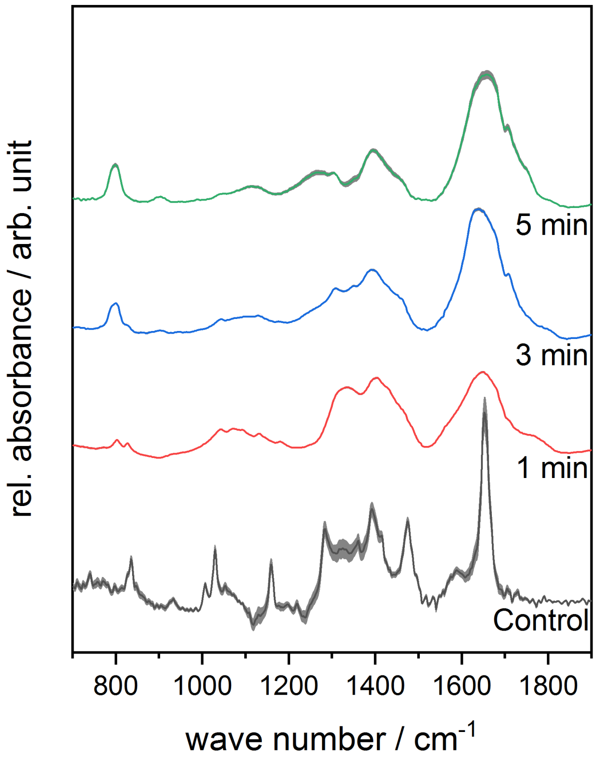

Figure S23. IR spectra of complex (**1**) after plasma treatment.

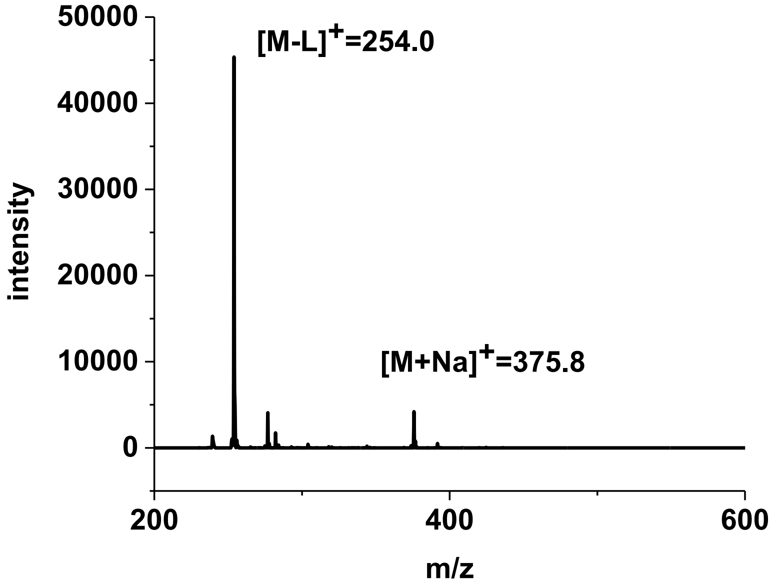

Figure S24. ESI-MS spectrum of complex (**2**) after 1 min of plasma treatment.

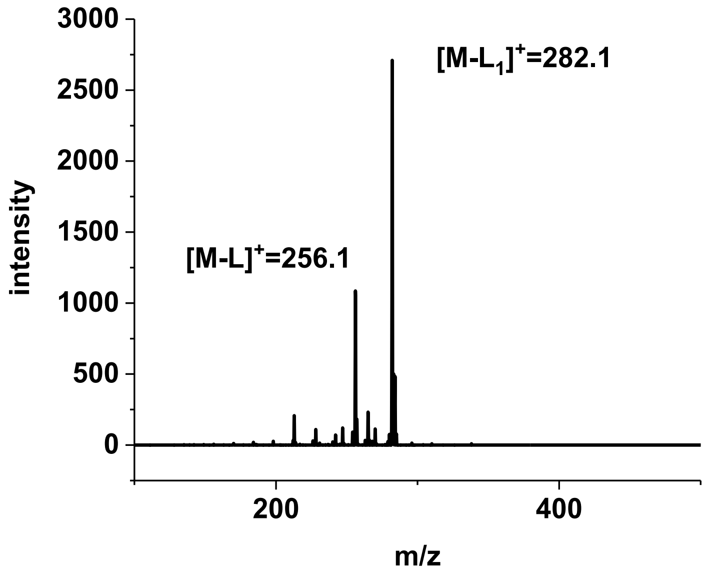

Figure S25. ESI-MS spectrum of complex (**2**) after 3 min of plasma treatment.

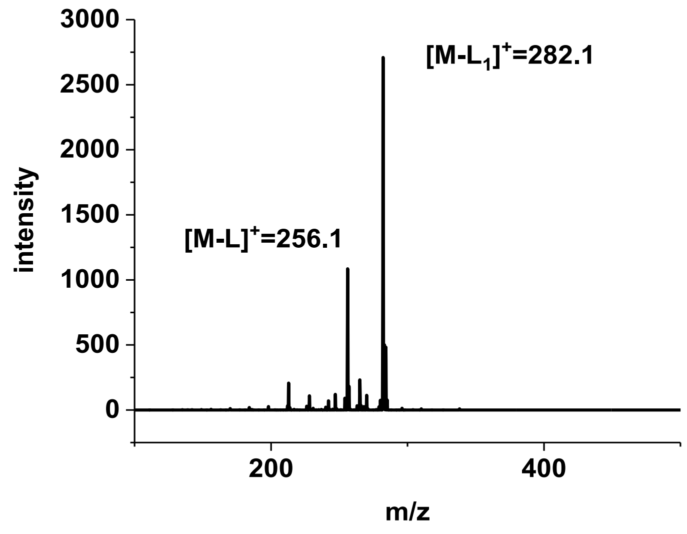

Figure S26. ESI-MS spectrum of complex (**2**) after 5 min of plasma treatment.

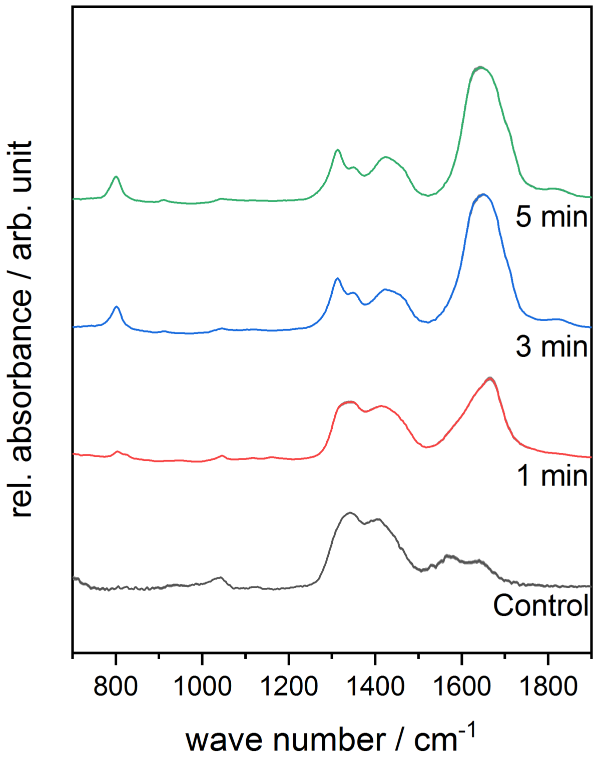

Figure S27. IR spectra of complex (**2**) after plasma treatment.

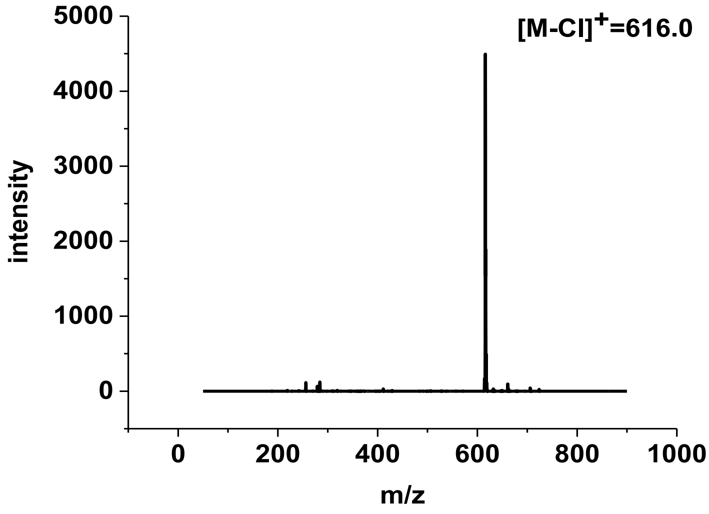

Figure S28. ESI-MS spectrum of complex (**3**) after 1 min of plasma treatment.

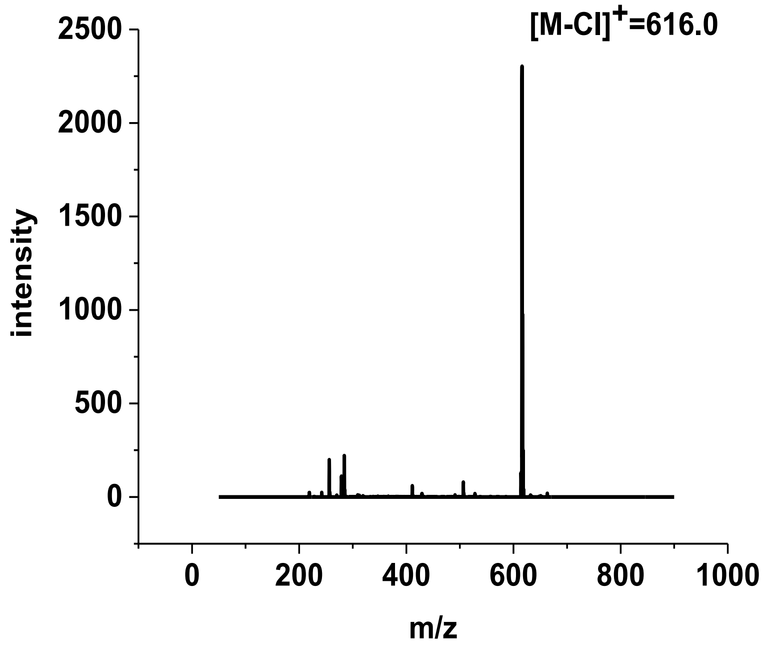

Figure S29. ESI-MS spectrum of complex (**3**) after 3 min of plasma treatment.

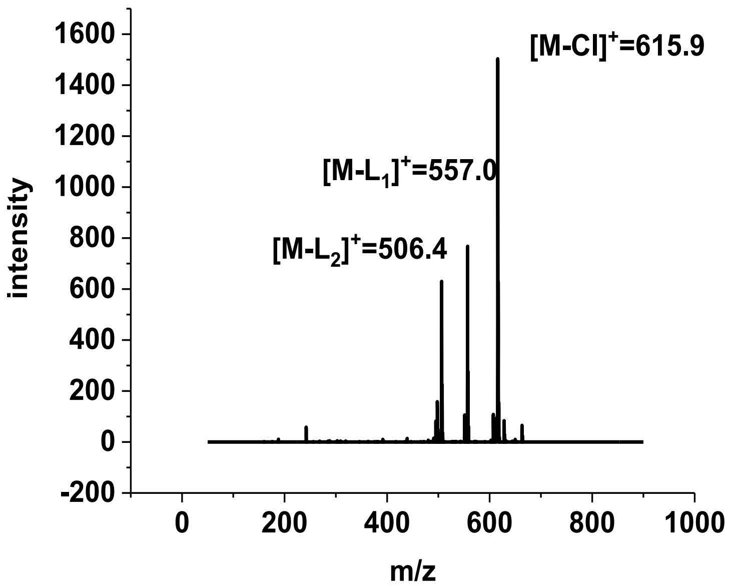

Figure S30. ESI-MS spectrum of complex (**3**) after 5 min of plasma treatment

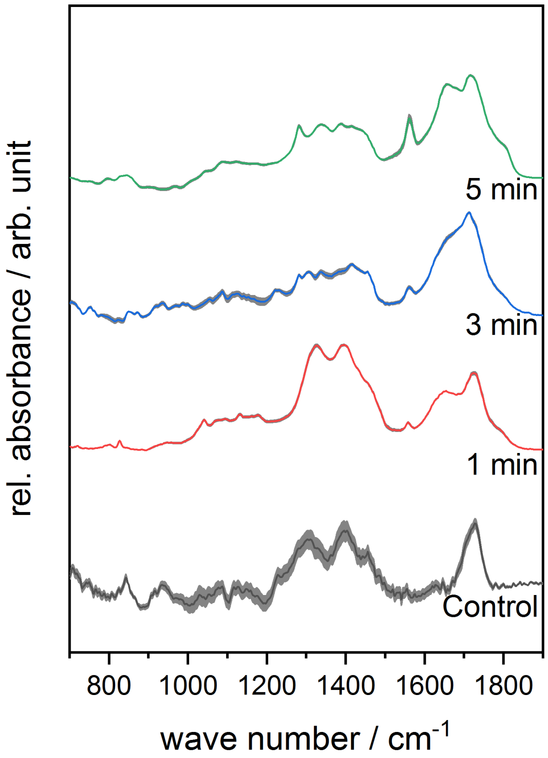

Figure S31. IR spectra of complex (**3**) after plasma treatment.

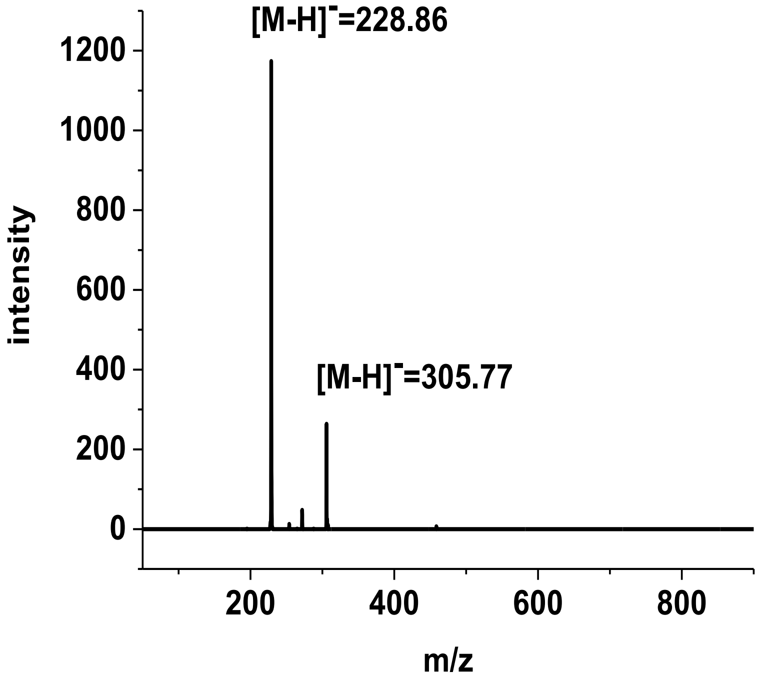

Figure S32. ESI-MS spectrum of complex (**1**) after 1 min of incubation with GSH.

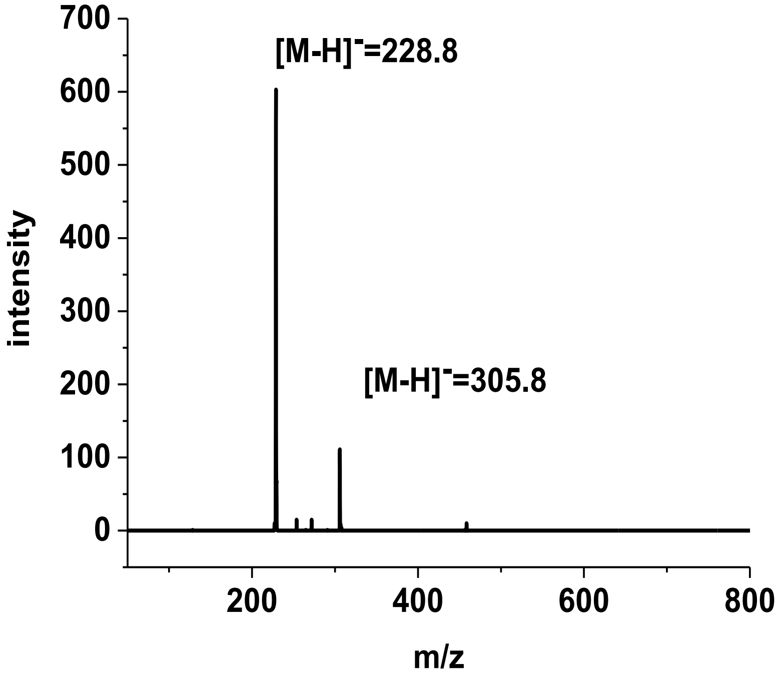

Figure S33. ESI-MS spectrum of complex (**1**) after 3 min of incubation with GSH.

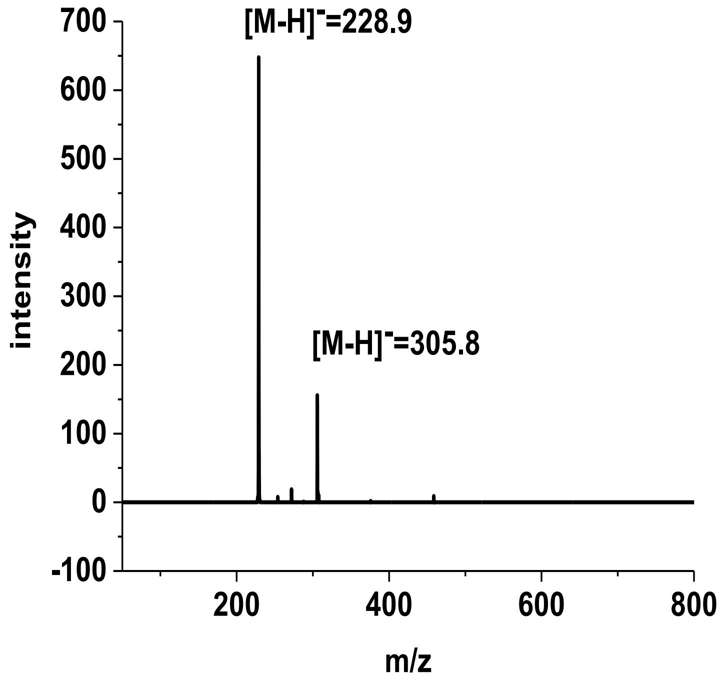

Figure S34. ESI-MS spectrum of complex (**1**) after 5 min of incubation with GSH.

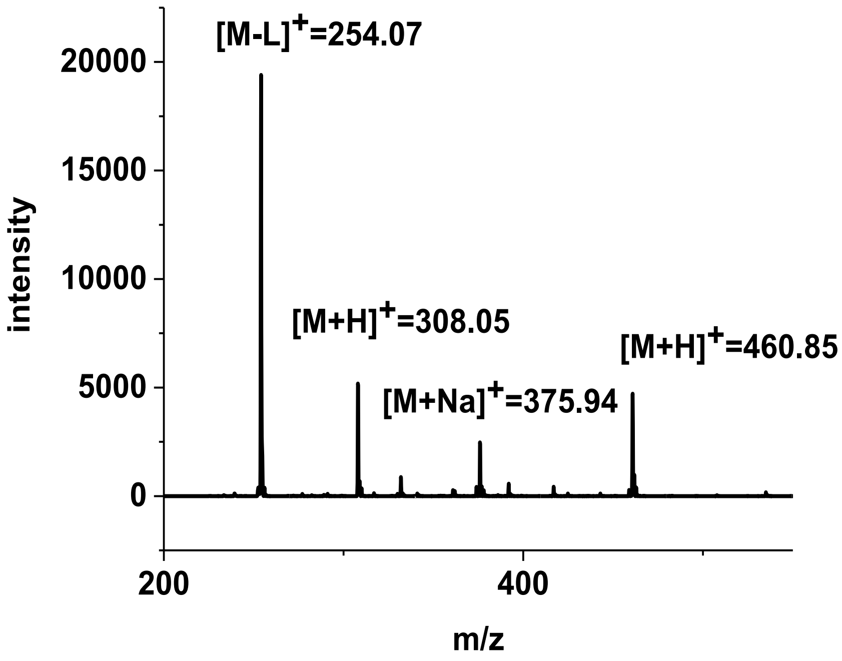

Figure S35. ESI-MS spectrum of complex (**2**) after 1 min of incubation with GSH.

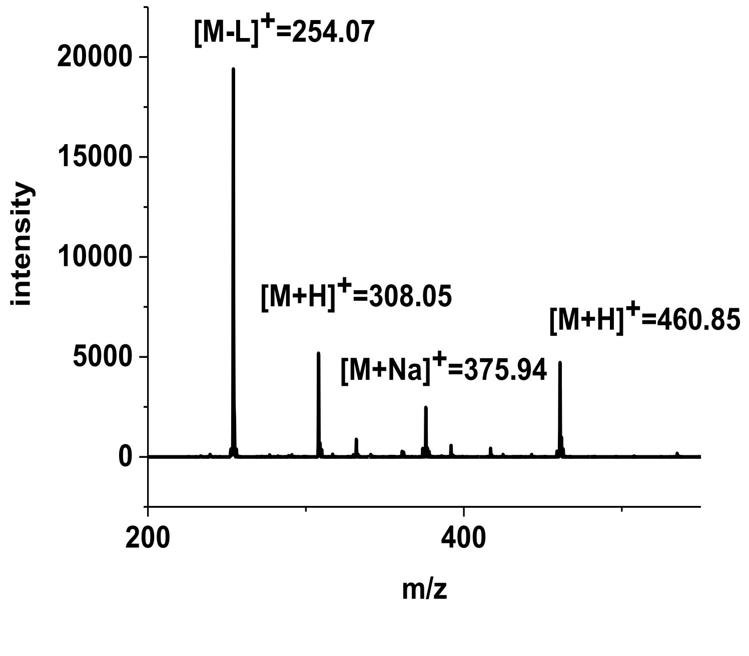

Figure S36. ESI-MS spectrum of complex (**2**) after 3 min of incubation with GSH.

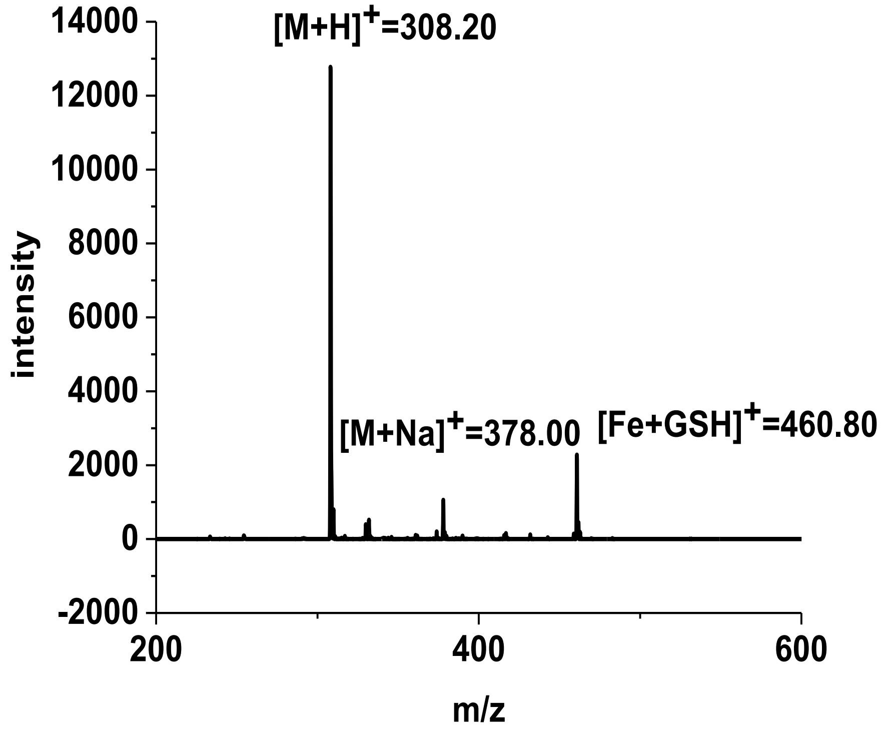

Figure S37. ESI-MS spectrum of complex (**2**) after 5 min of incubation with GSH.

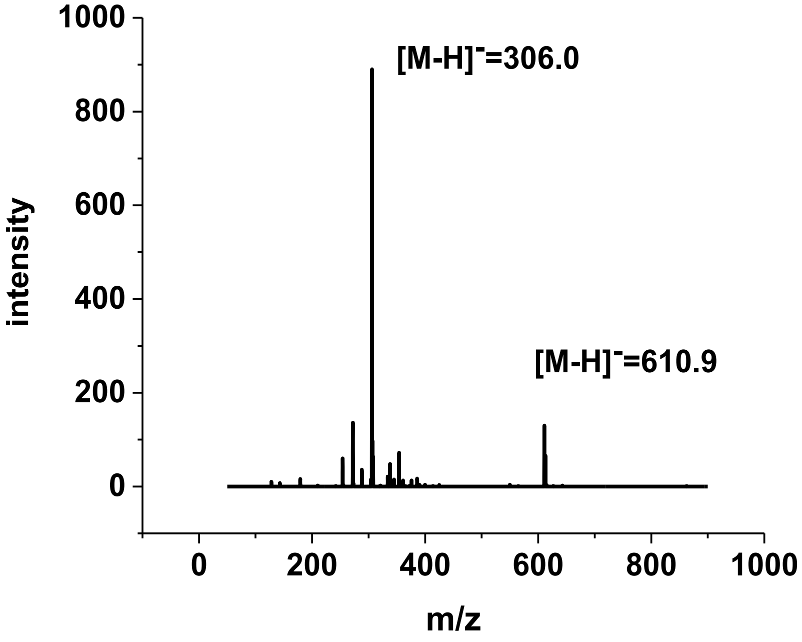

Figure S38. ESI-MS spectrum of complex (**3**) after 1 min of incubation with GSH.

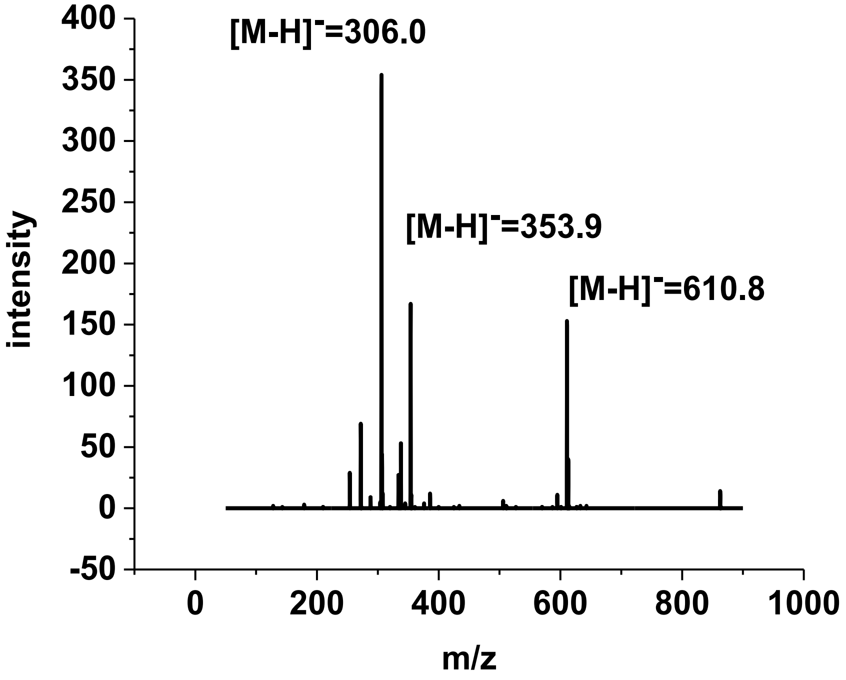

Figure S39. ESI-MS spectrum of complex (**3**) after 3 min of incubation with GSH.

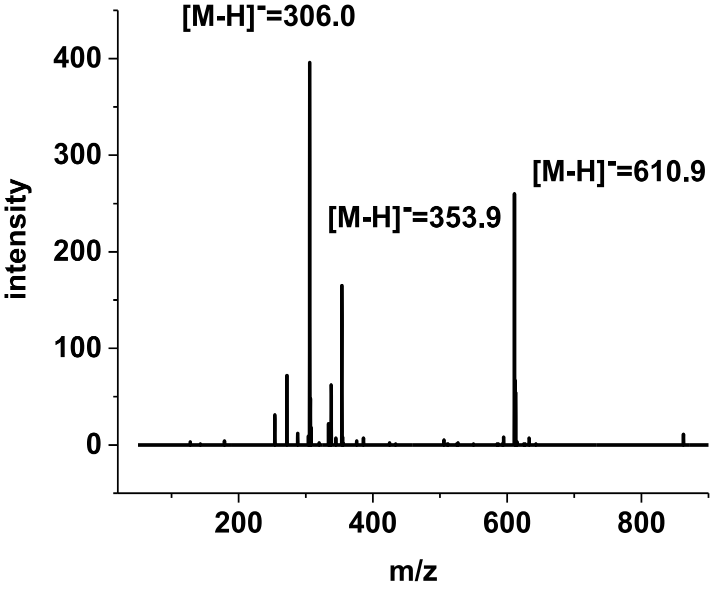

Figure S40. ESI-MS spectrum of complex (**3**) after 5 min of incubation with GSH.

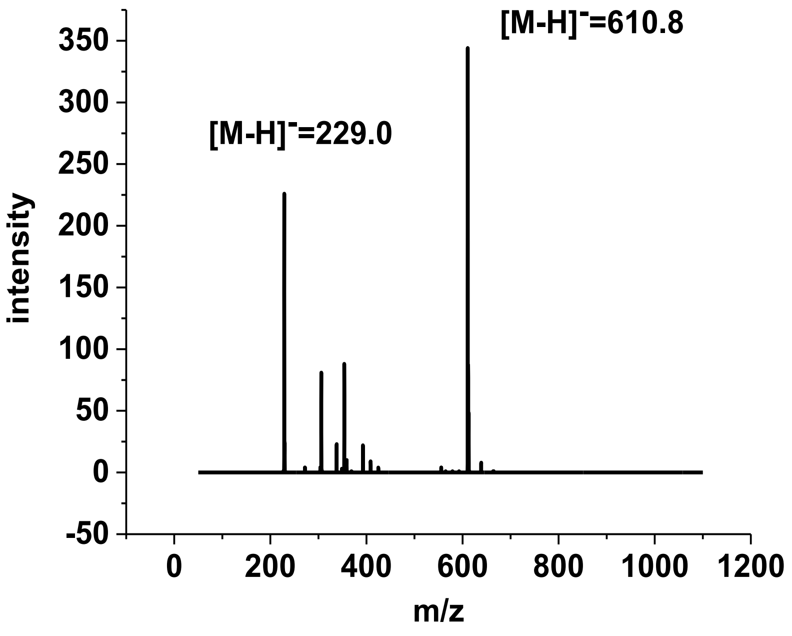

Figure S41. ESI-MS spectrum of complex (**1**) after 1 min of incubation with GSSG.

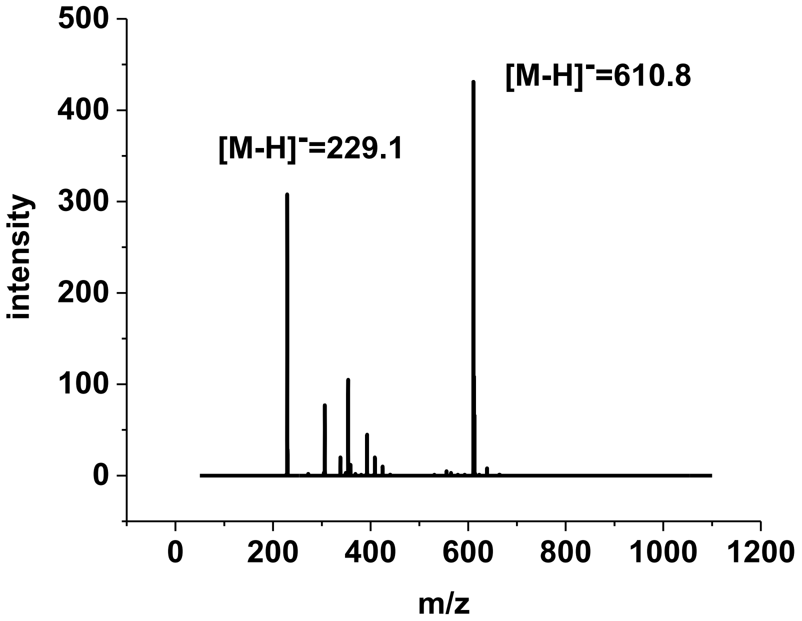

Figure S42. ESI-MS spectrum of complex (**1**) after 3 min of incubation with GSSG.

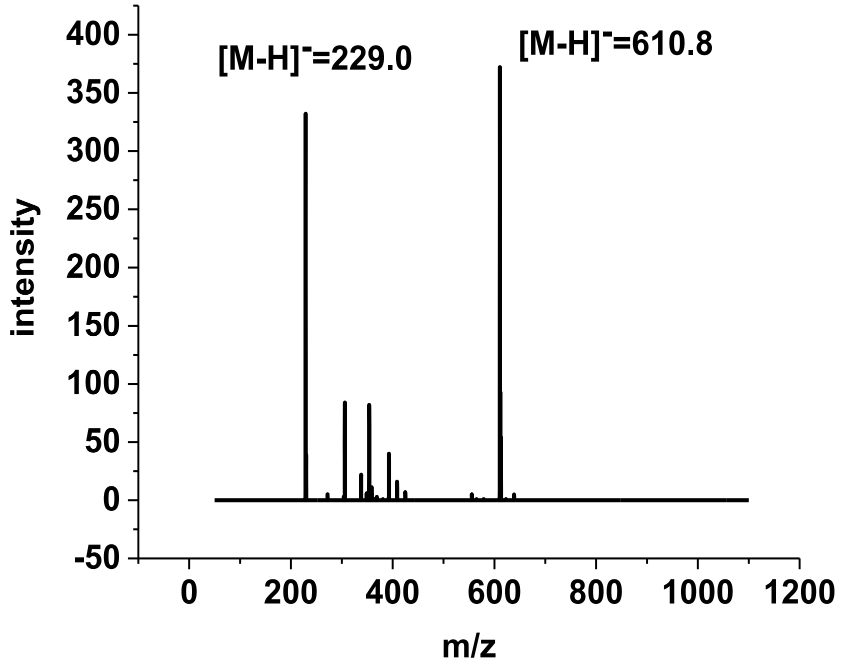

Figure S43. ESI-MS spectrum of complex (**1**) after 5 min of incubation with GSSG.

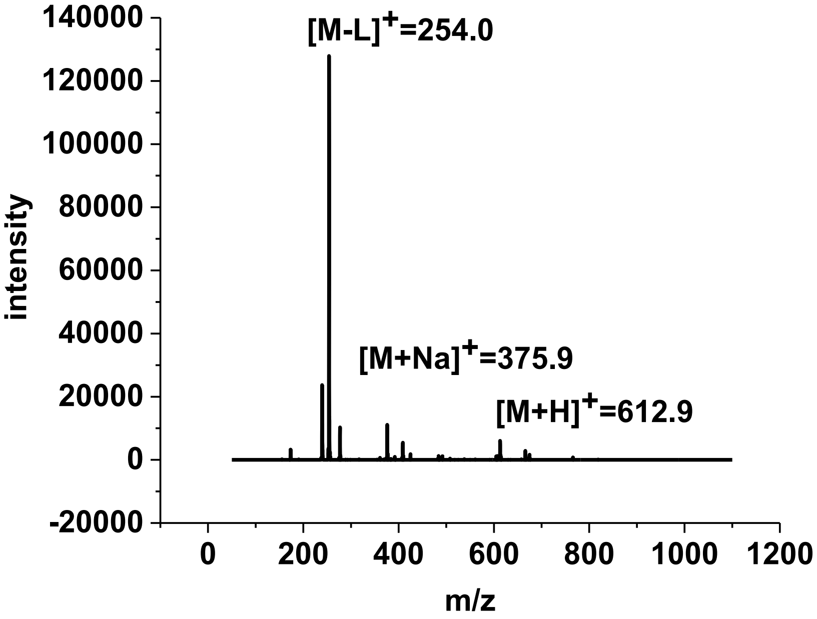

Figure S44. ESI-MS spectrum of complex (**2**) after 1 min of incubation with GSSG.

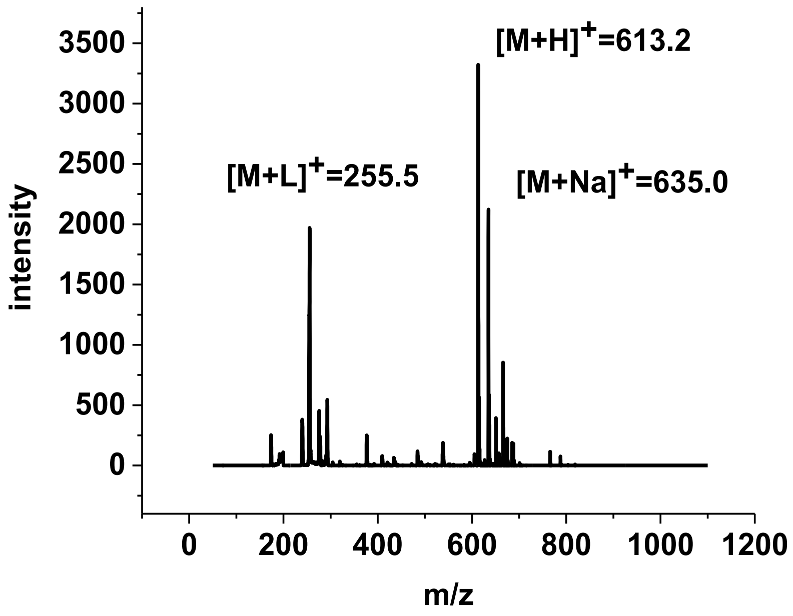

Figure S45. ESI-MS spectrum of complex (**2**) after 3 min of incubation with GSSG.

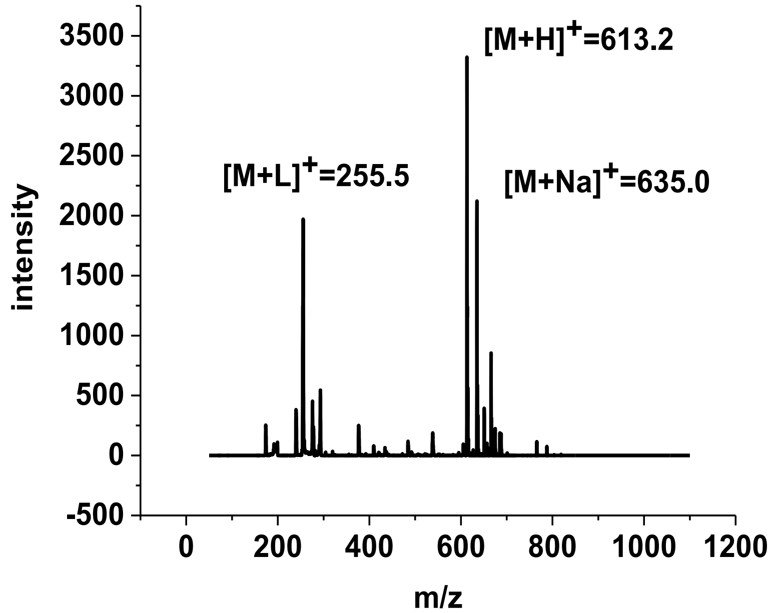

Figure S46. ESI-MS spectrum of complex (**2**) after 5 min of incubation with GSSG.

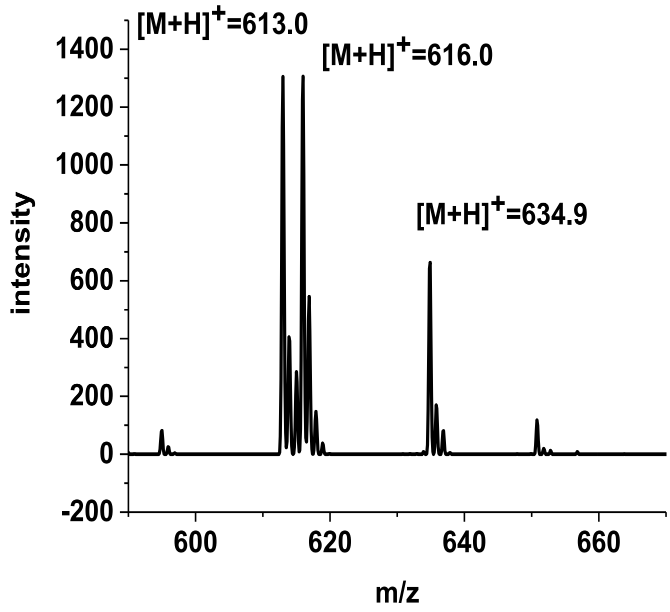

Figure S47. ESI-MS spectrum of complex (**3**) after 1 min of incubation with GSSG.

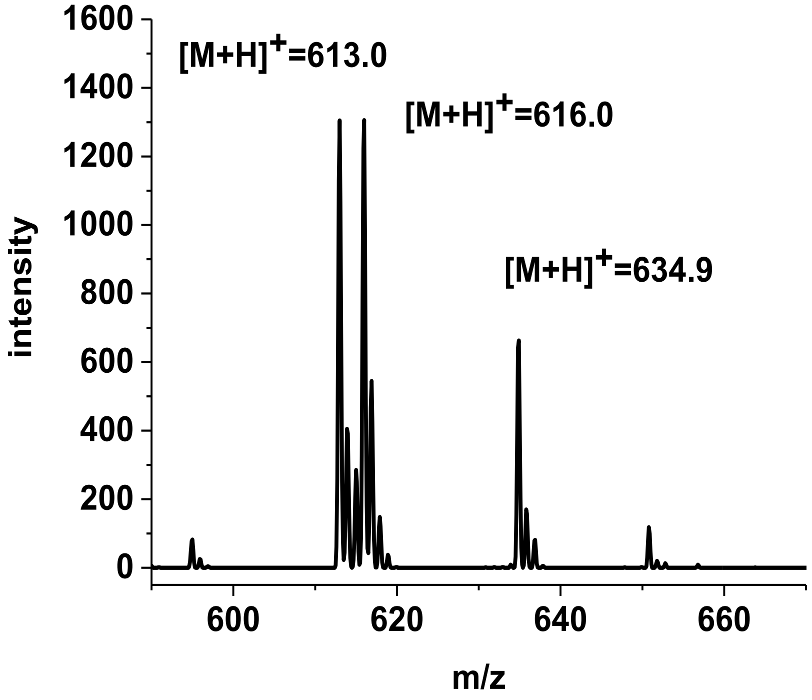

Figure S48. ESI-MS spectrum of complex (**3**) after 3 min of incubation with GSSG.

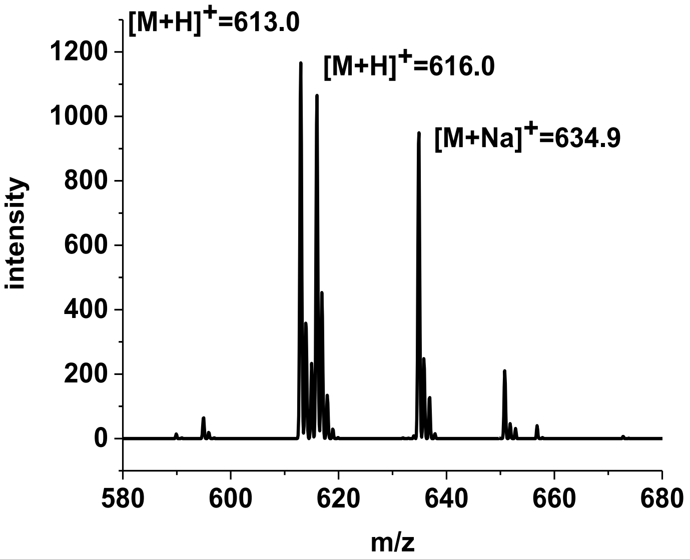

Figure S49. ESI-MS spectrum of complex (**3**) after 5 min of incubation with GSSG.

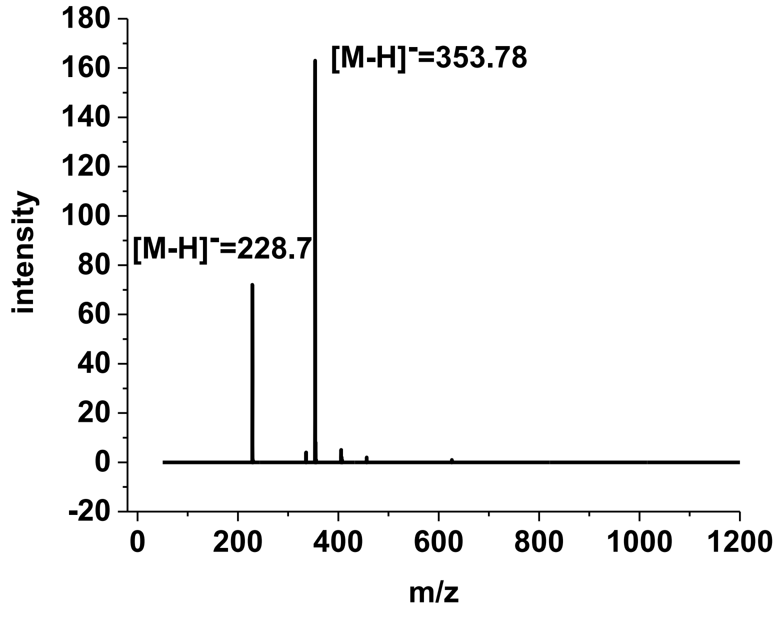

Figure S50. ESI-MS spectrum of GSH after 1 min of plasma treatment in the presence of complex (**1**)

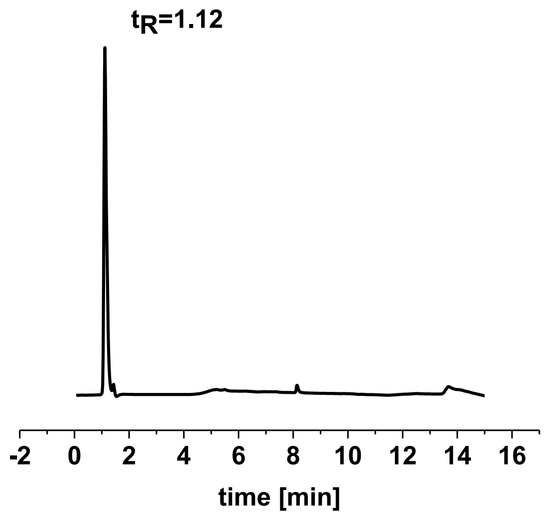

Figure S51. HPLC chromatogram GSH after 1 min of plasma treatment in the presence of complex (**1**).

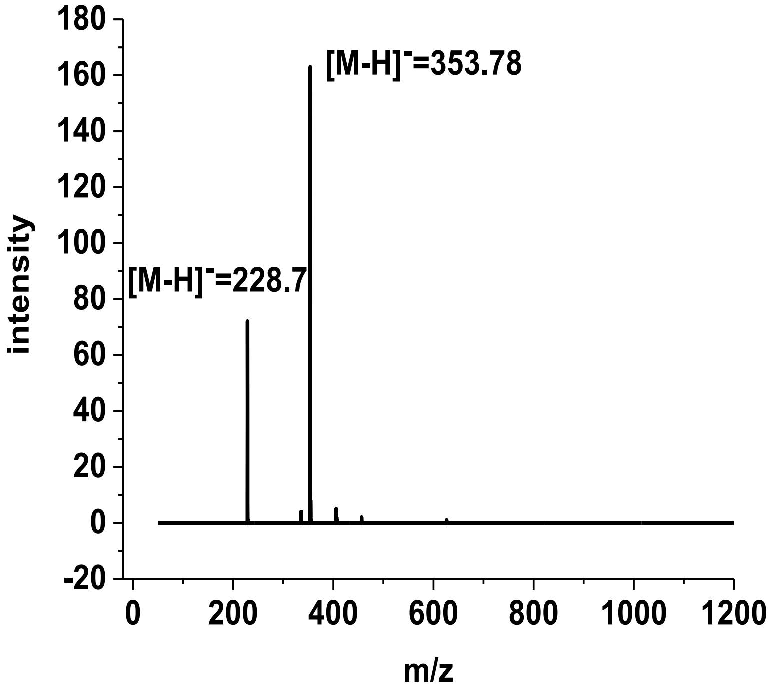

Figure S52. ESI-MS spectrum of GSH after 3 min of plasma treatment in the presence of complex (**1**)

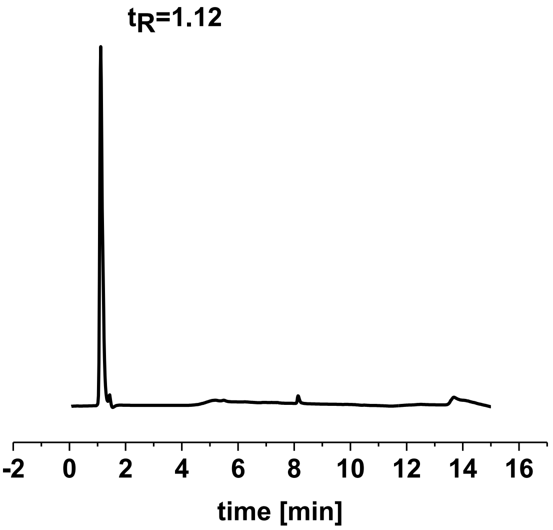

Figure S53. HPLC chromatogram GSH after 3 min of plasma treatment in the presence of complex (**1**).

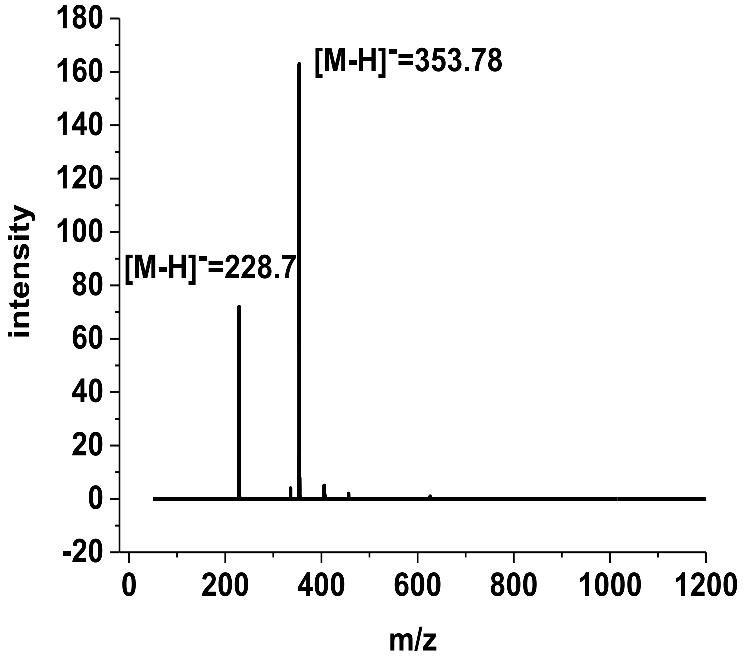

Figure S54. ESI-MS spectrum of GSH after 5 min of plasma treatment in the presence of complex (**1**)

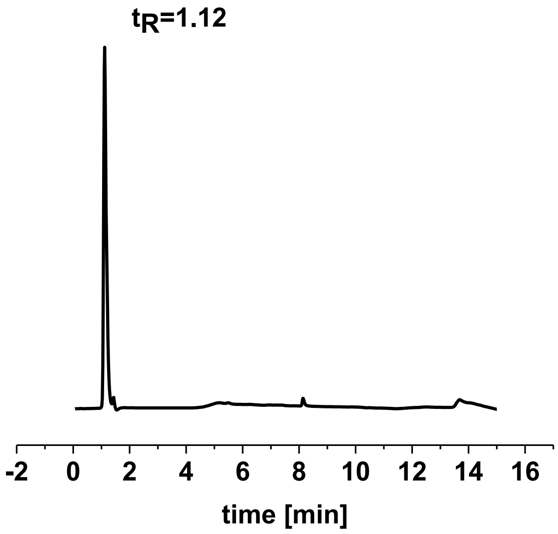

Figure S55. HPLC chromatogram GSH after 5 min of plasma treatment in the presence of complex (**1**).

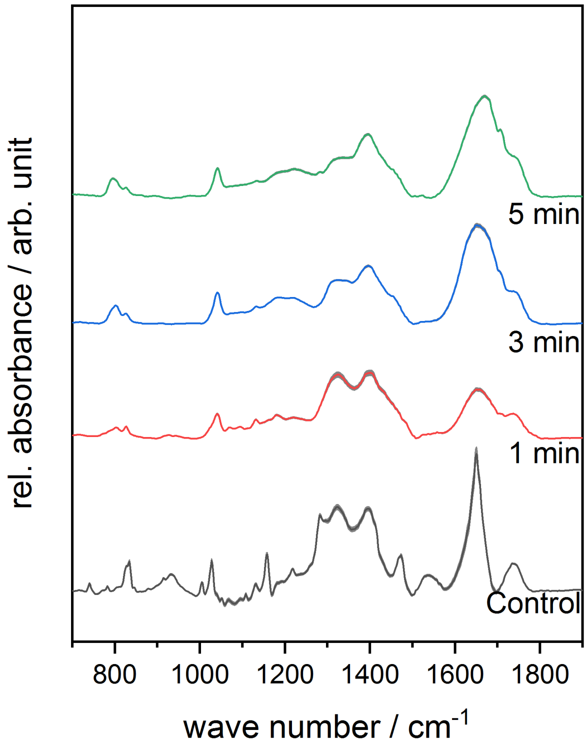

Figure S56. IR spectra of GSH after 5 min of plasma treatment in the presence of complex (**1**).

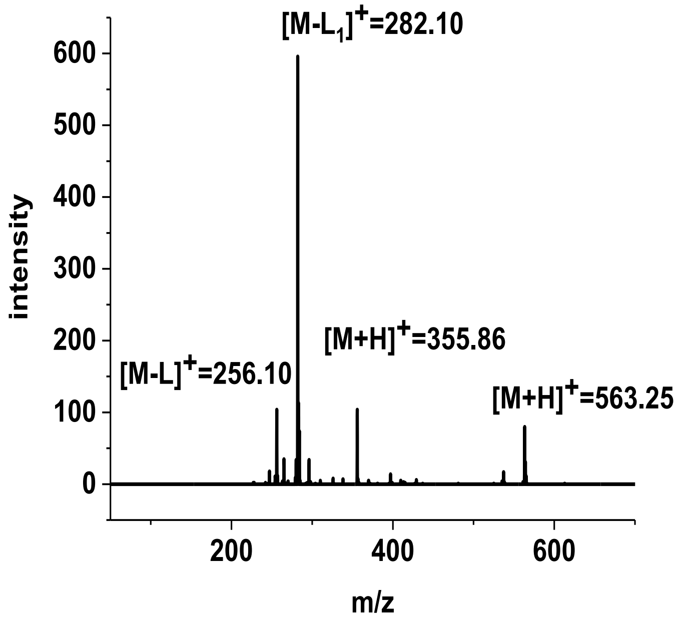

Figure S57. ESI-MS spectrum of GSH after 1 min of plasma treatment in the presence of complex (**2**)

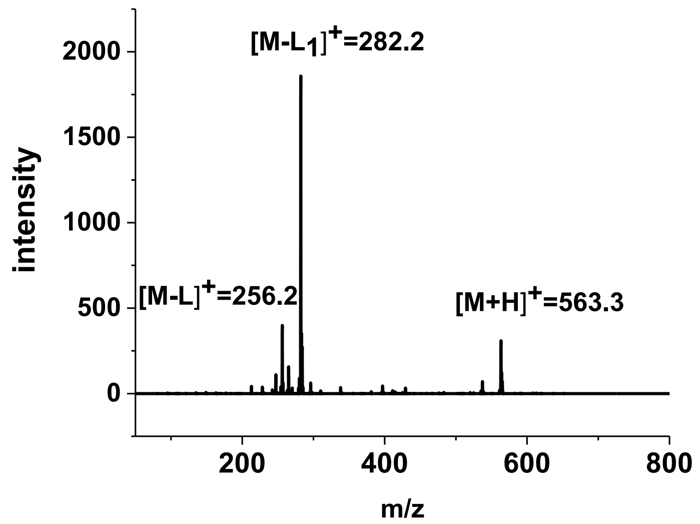

. **Figure S58.** ESI-MS spectrum of GSH after 3 min of plasma treatment in the presence of complex (**2**).

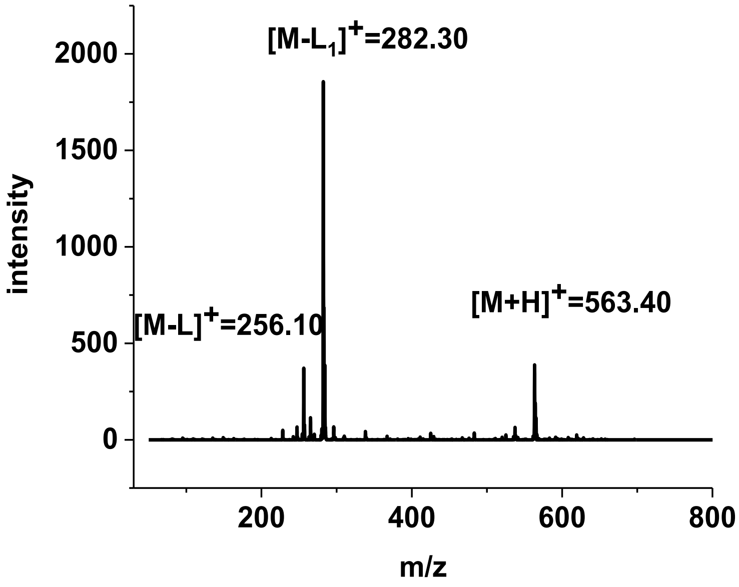

Figure S59. ESI-MS spectrum of GSH after 5 min of plasma treatment in the presence of complex (**2**)

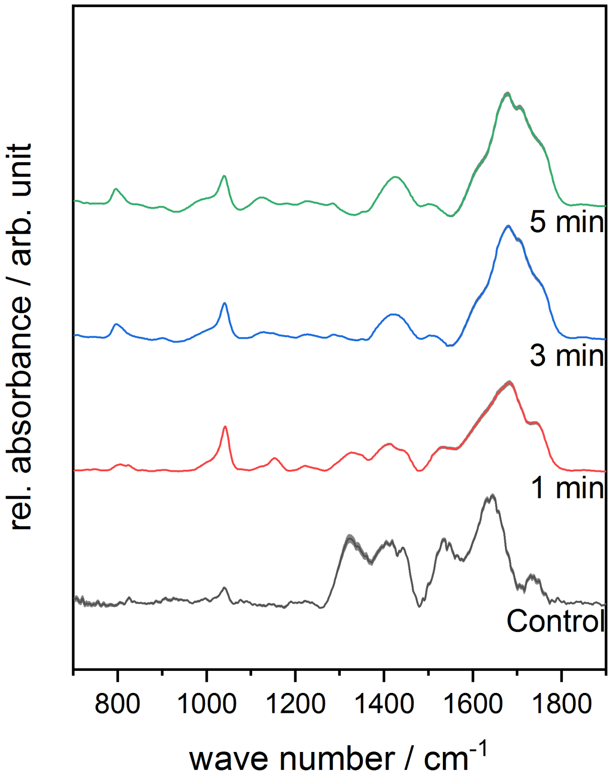

Figure S60. IR spectra of GSH after plasma treatment in the presence of complex (**2**).

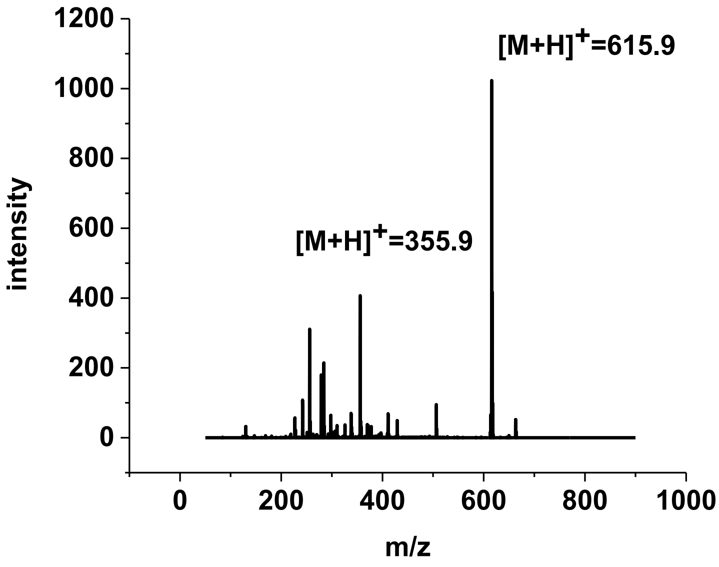

Figure S61. ESI-MS spectrum of GSH after 1 min of plasma treatment in the presence of complex (**3**)

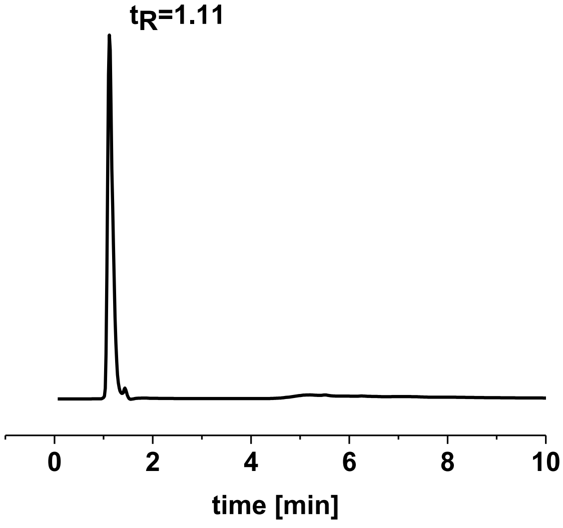

Figure S62. HPLC chromatogram GSH after 1 min of plasma treatment in the presence of complex (**3**).

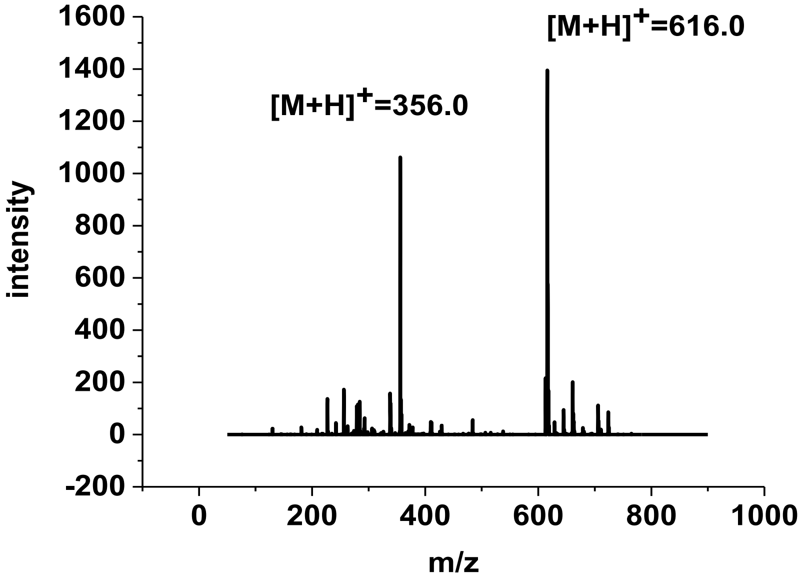

Figure S63. ESI-MS spectrum of GSH after 3 min of plasma treatment in the presence of complex (**3**).

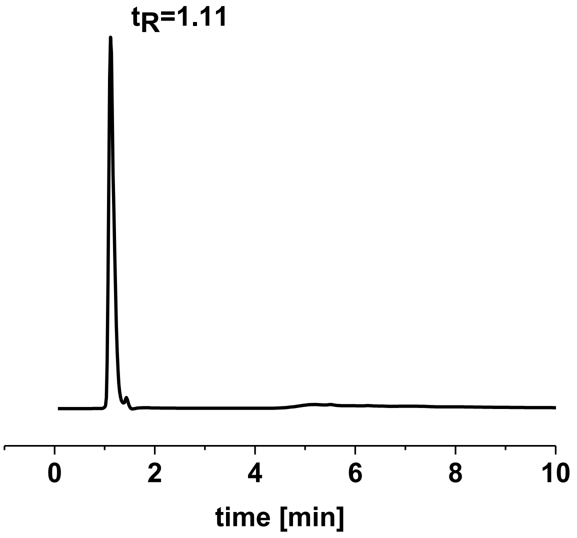

Figure S64. HPLC chromatogram GSH after 3 min of plasma treatment in the presence of complex (**3**).

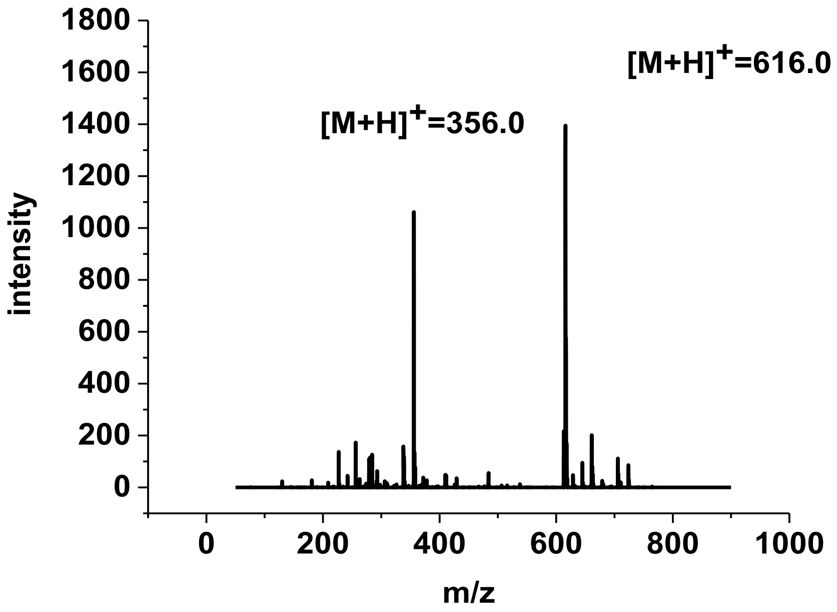

Figure S65. ESI-MS spectrum of GSH after 5 min of plasma treatment in the presence of complex (**3**)

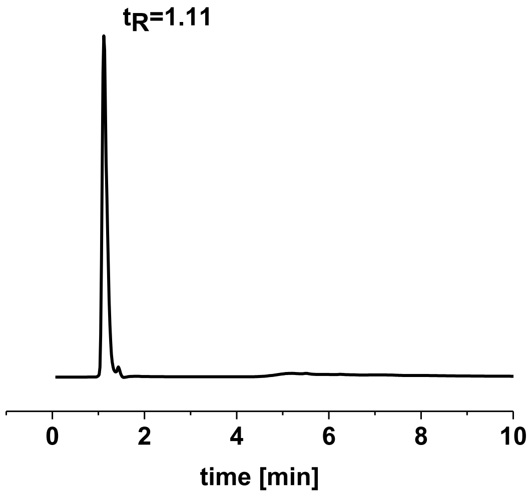

Figure S66. HPLC chromatogram GSH after 5 min of plasma treatment in the presence of complex (**3**).

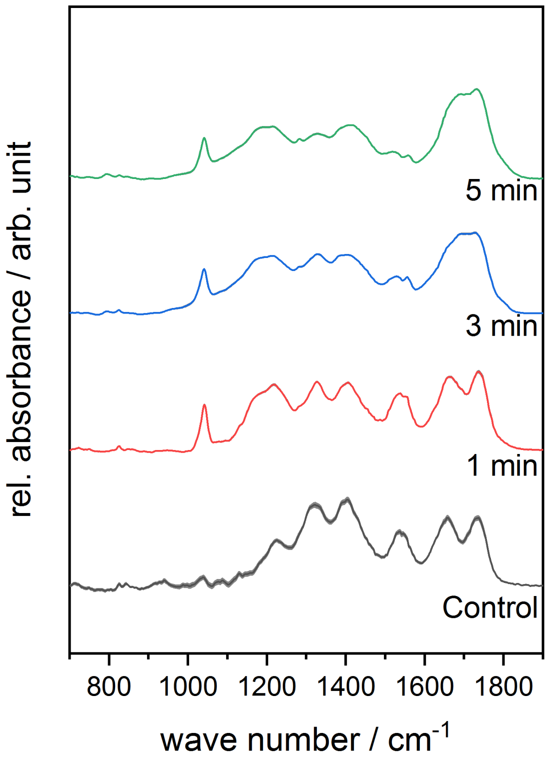

Figure S67. IR spectra of GSH after plasma treatment in the presence of complex (**3**).

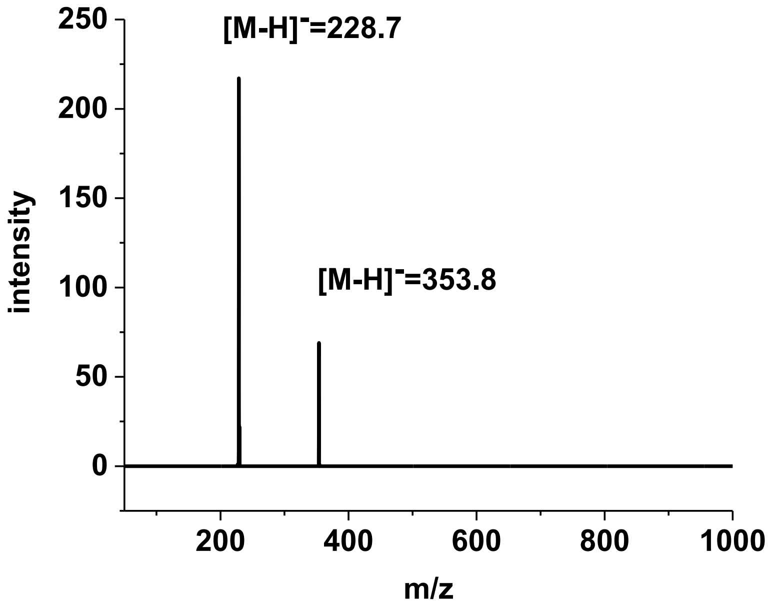

Figure S68. ESI-MS spectrum of GSSG after 1 min of plasma treatment in the presence of complex (**1**).

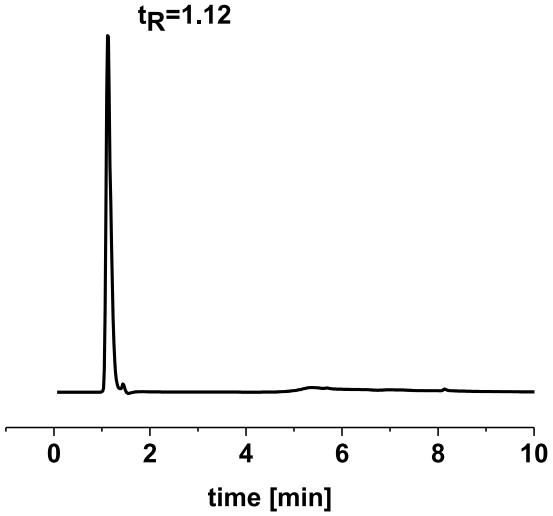

Figure S69. HPLC chromatogram GSSG after 1 min of plasma treatment in the presence of complex (**1**).

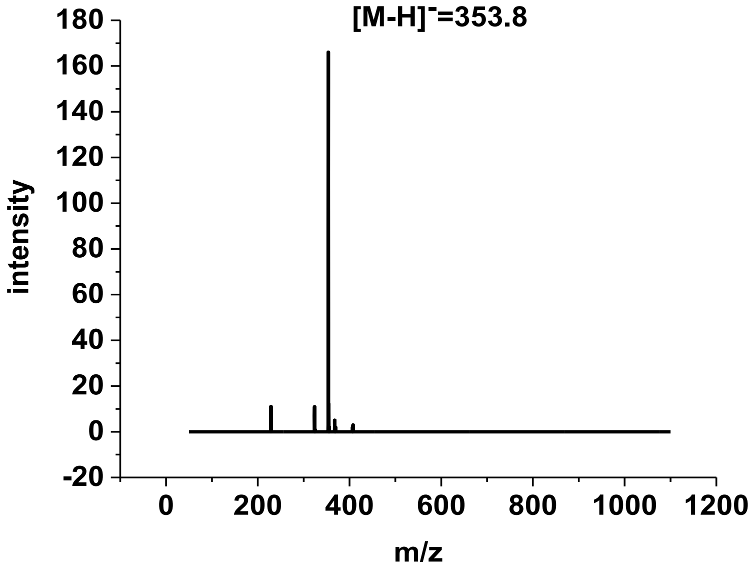

Figure S70. ESI-MS spectrum of GSSG after 3 min of plasma treatment in the presence of complex (**1**).

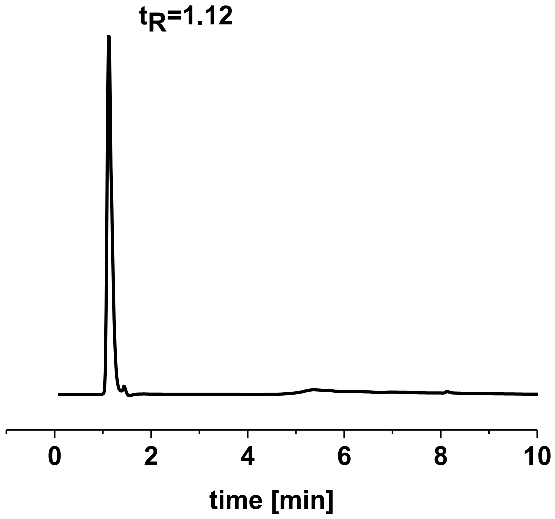

Figure S71. HPLC chromatogram GSSG after 3 min of plasma treatment in the presence of complex (**1**).

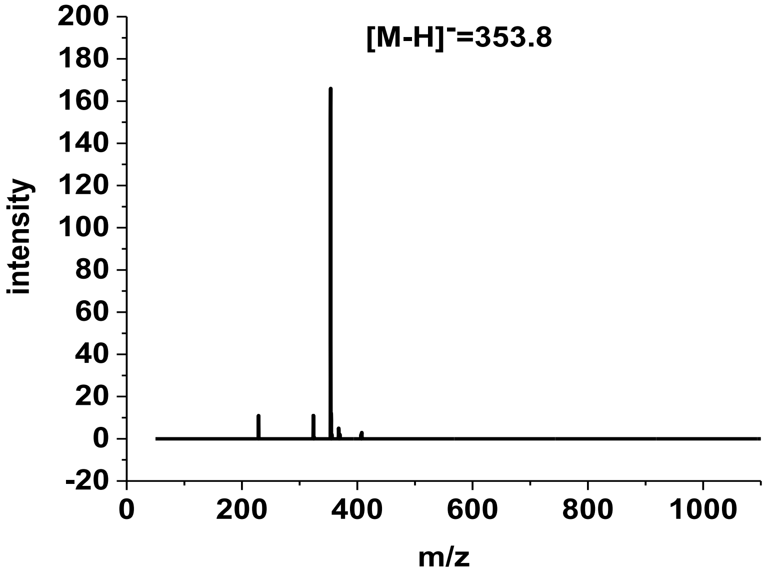

Figure S72. ESI-MS spectrum of GSSG after 5 min of plasma treatment in the presence of complex (**1**).

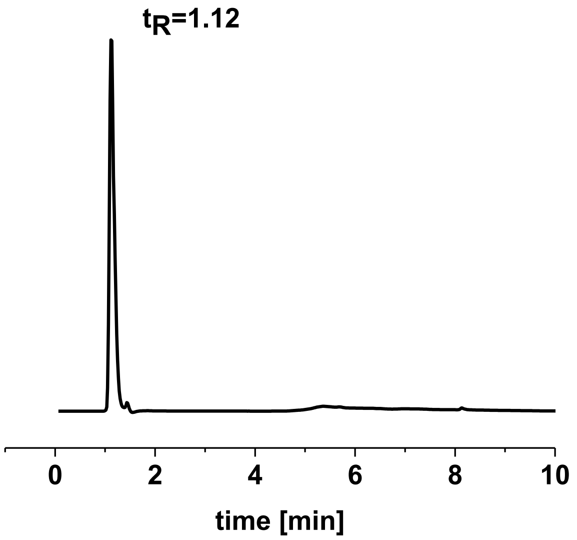

Figure S73. HPLC chromatogram GSSG after 5 min of plasma treatment in the presence of complex (**1**).

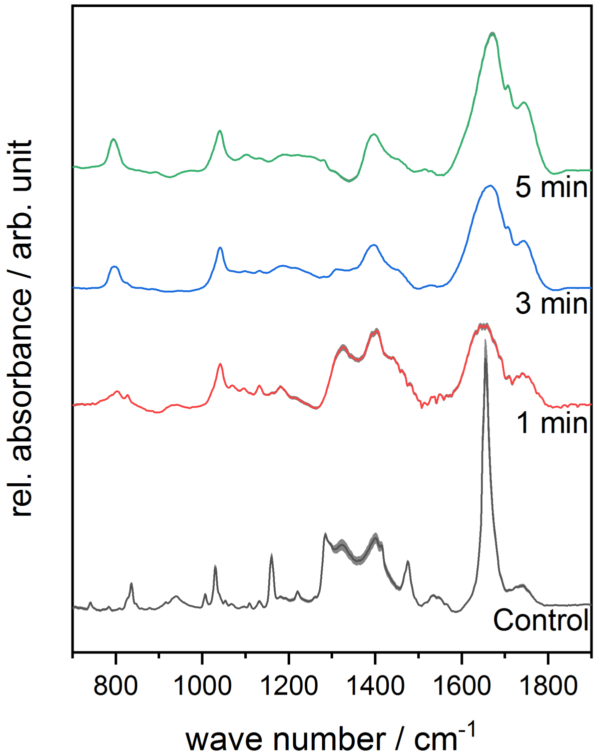

Figure S74. IR spectra of GSSG after plasma treatment in the presence of complex (**1**).

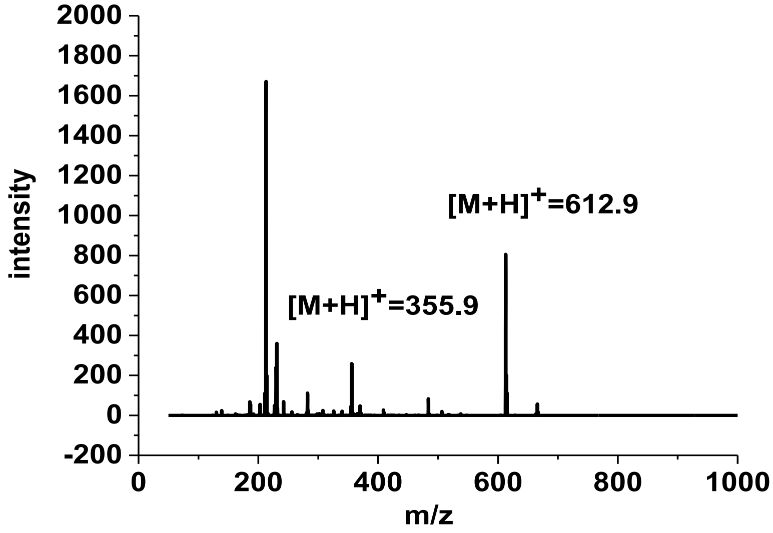

Figure S75. ESI-MS spectrum of GSSG after 1 min of plasma treatment in the presence of complex (**2**).

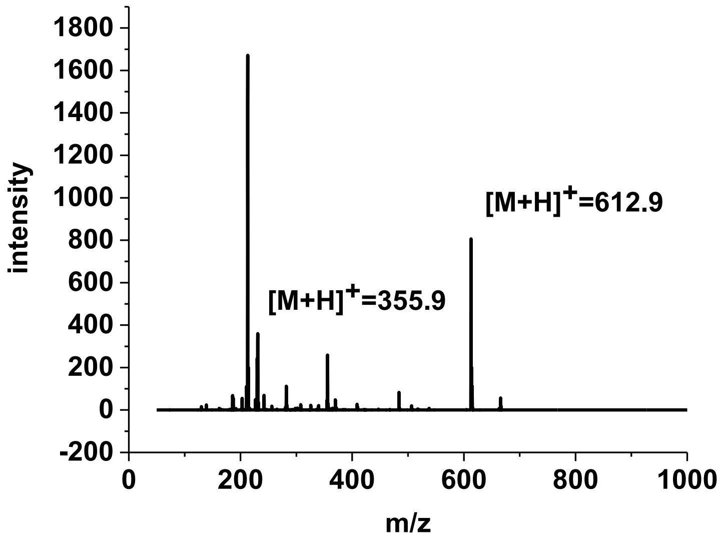

Figure S76. ESI-MS spectrum of GSSG after 3 min of plasma treatment in the presence of complex (**2**).

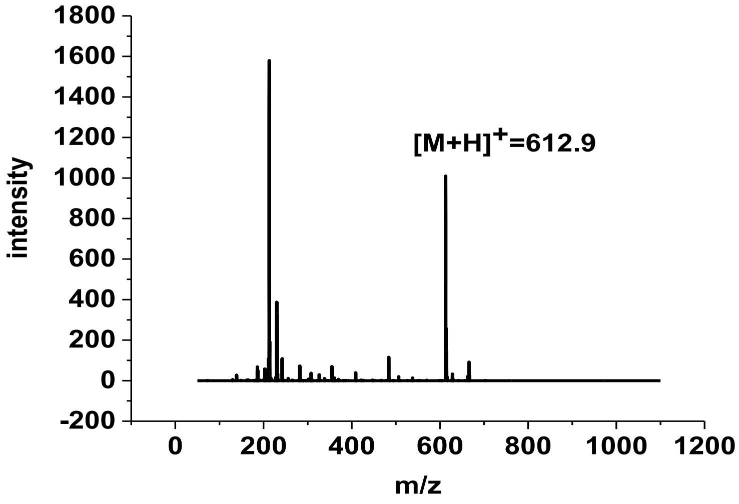

Figure S77. ESI-MS spectrum of GSSG after 5 min of plasma treatment in the presence of complex (**2**).

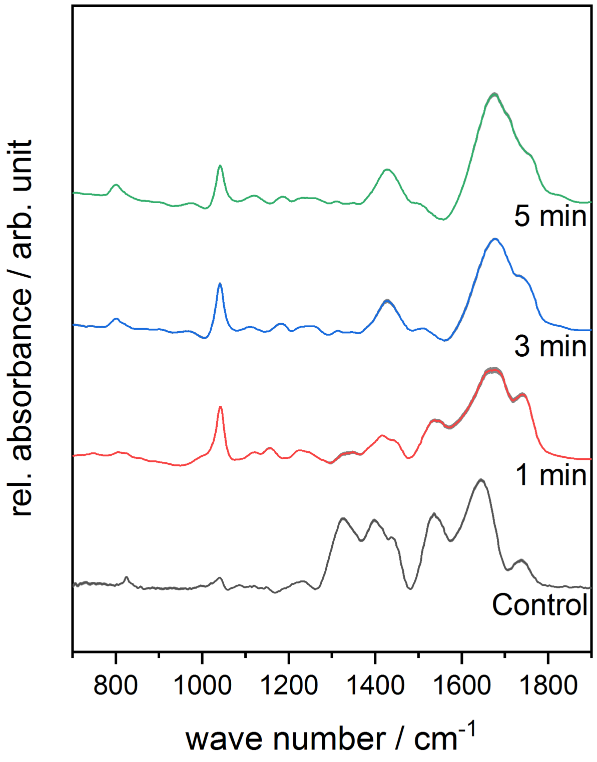

Figure S78. IR spectra of GSSG after plasma treatment in the presence of complex (**2**).

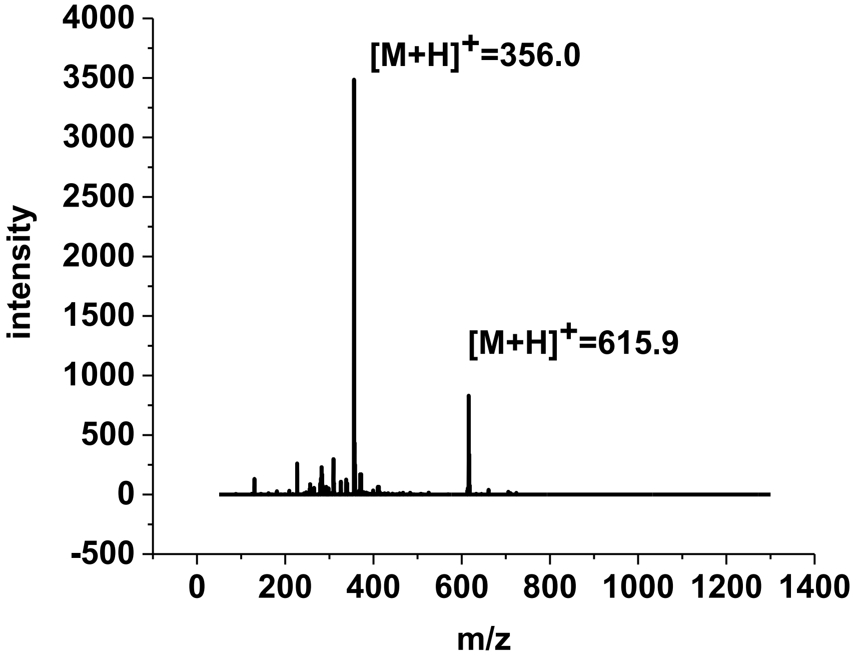

Figure S79. ESI-MS spectrum of GSSG after 1 min of plasma treatment in the presence of complex (**3**).

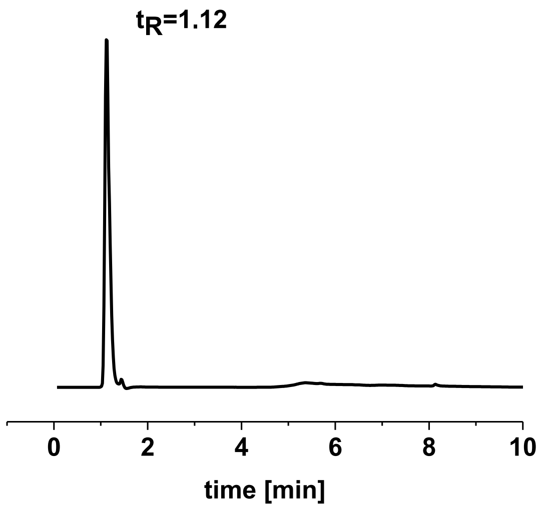

Figure S80. HPLC chromatogram GSSG after 1 min of plasma treatment in the presence of complex (**3**).

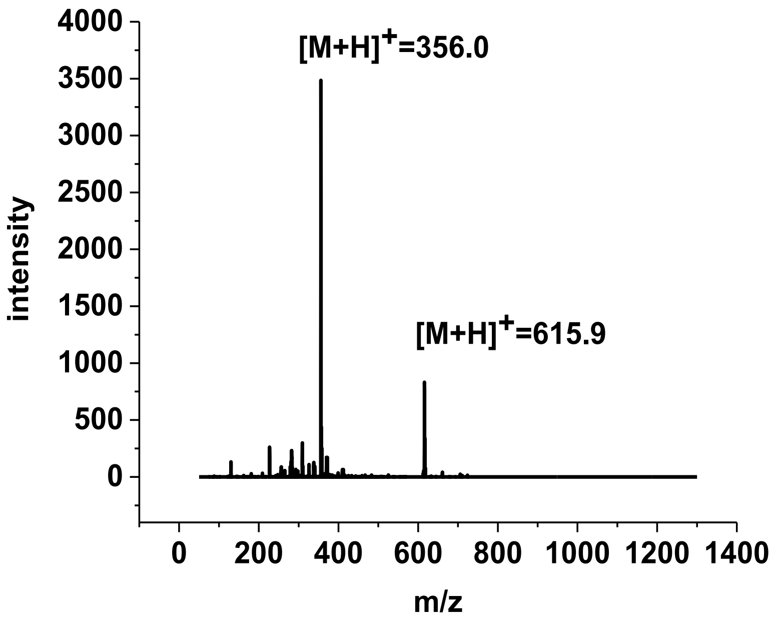

Figure S81. ESI-MS spectrum of GSSG after 3 min of plasma treatment in the presence of complex (**3**).

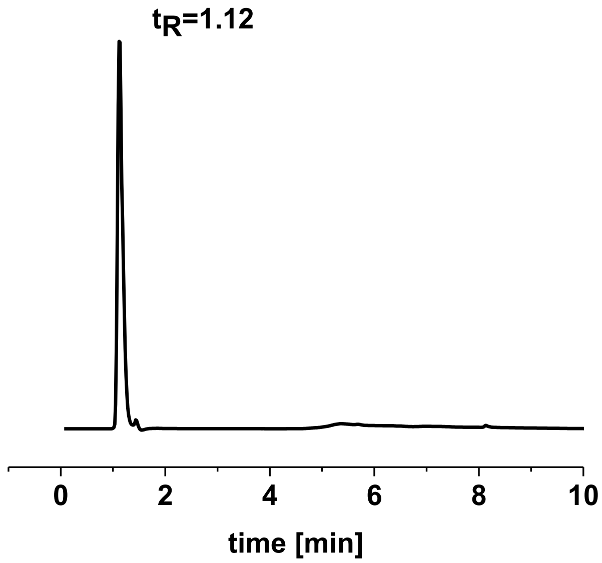

Figure S82. HPLC chromatogram GSSG after 3 min of plasma treatment in the presence of complex (**3**).

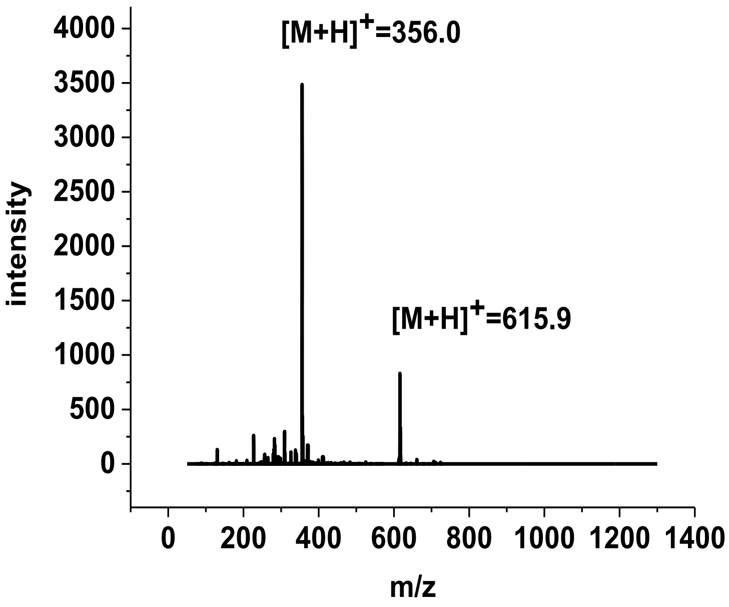

Figure S83. ESI-MS spectrum of GSSG after 5 min of plasma treatment in the presence of complex (**3**).

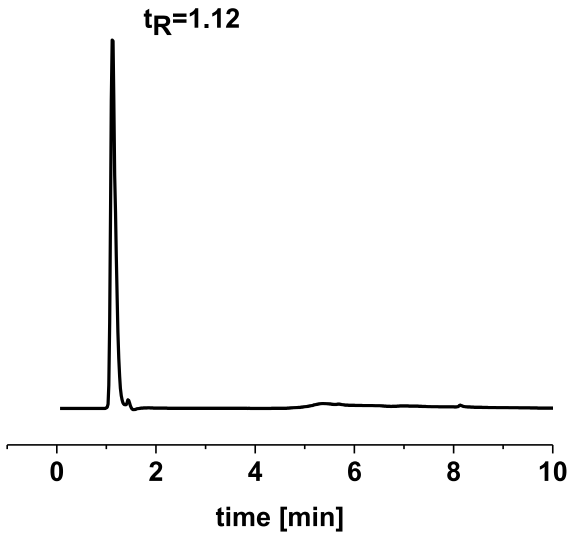

Figure S84. HPLC chromatogram GSSG after 5 min of plasma treatment in the presence of complex (**3**).

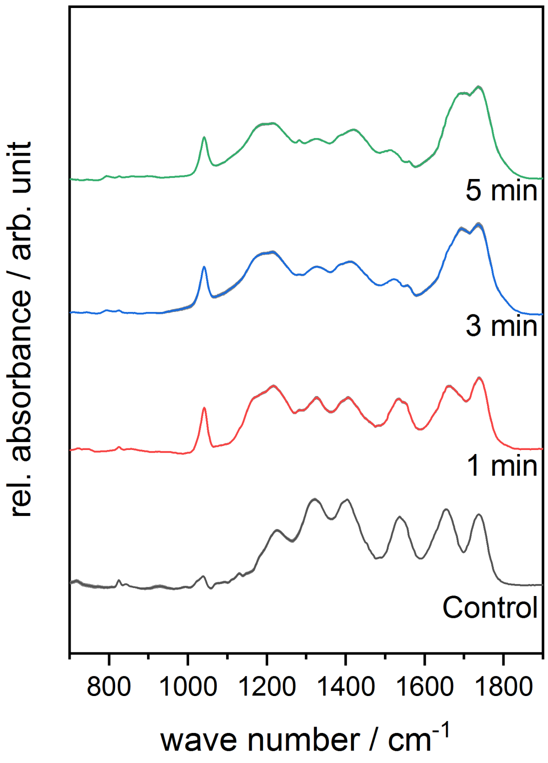

Figure S85. IR spectra of GSSG after plasma treatment in the presence of complex (**3**).

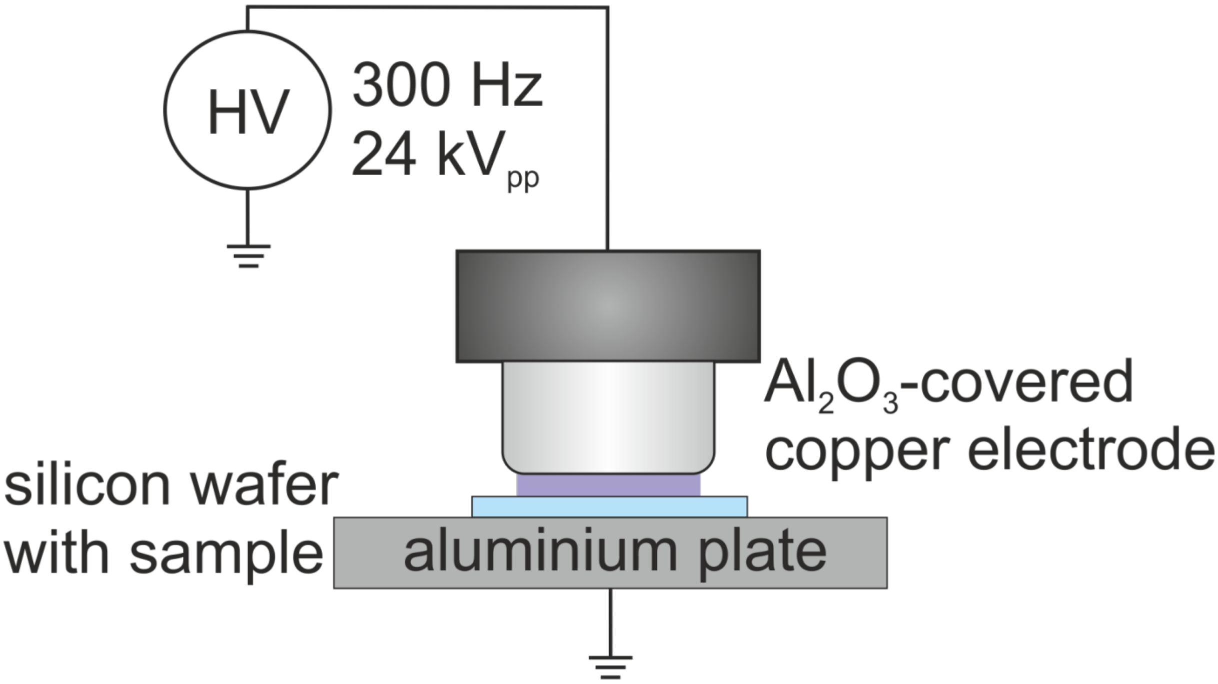

Figure S86. The scheme of the plasma source. [Kogelheide, F. et al. FT-IR spectroscopy of cysteine as a ready-to-use method for the investigation of plasma-induced chemical modifications of macromolecules. J. Phys. D: Appl. Phys. 49, 084004, DOI: https://doi.org/10.1088/0022-3727/49/8/084004 (2016).]
